# Influence of infant fucosyltransferase polymorphisms on the association of maternal secretor status with child outcomes: the Ulm birth cohort studies

**DOI:** 10.1038/s41598-025-28217-w

**Published:** 2025-11-18

**Authors:** Linda P. Siziba, Marko Mank, Bernd Stahl, Hermann Brenner, John Gonsalves, Bernadet Blijenberg, Katrin Horn, Markus Scholz, Parastoo Kheiroddin, Michael Kabesch, Deborah Wernecke, Dietrich Rothenbacher, Jon Genuneit

**Affiliations:** 1 Pediatric Epidemiology, Department of Paediatrics, Medical Faculty, Leipzig University, 04103 Leipzig, Germany; 2 Danone Research & Innovation, Utrecht, 3584 The Netherlands; 3 Department of Chemical Biology & Drug Discovery, Utrecht Institute for Pharmaceutical Sciences, Utrecht University, Utrecht, 3584 The Netherlands; 4 Division of Clinical Epidemiology and Aging Research, German Cancer Research Centre (DKFZ), Im Neuenheimer Feld 581, 69120 Heidelberg, Germany; 5 Institute for Medical Informatics, Statistics and Epidemiology (IMISE), Medical Faculty, Leipzig University, 04107 Leipzig, Saxony Germany; 6 Department of Pediatric Pneumology and Allergy, University Children’s Hospital Regensburg (KUNO), University of Regensburg and The Order of St. John at the St. Hedwig Hospital, Regensburg, Germany; 7 Institute of Epidemiology and Medical Biometry, Ulm University, 89075 Ulm, Germany; 8 German Center for Child and Adolescent Health (DZKJ), Partner Site Ulm, Ulm, Germany

**Keywords:** Fucosyltransferase (FUT2), Secretor milk, Non-secretor milk, Single nucleotide polymorphisms (SNPs), Otitis media (OM), Lower respiratory tract infections (LRTI), Upper respiratory tract infections (URTI), Body mass index (BMI), Genetics, Immunology, Diseases, Medical research

## Abstract

The influence of maternal secretor status and child fucosyltransferase (FUT2) polymorphisms on respiratory and ear infections, and growth, is an emerging research area. But, the impact of discordant secretor status between mother and infant, where their secretor status differs, remains underexplored. This study investigated how infant FUT2 single nucleotide polymorphisms (SNPs) modify the association between maternal secretor/non-secretor milk phenotypes and child outcomes such as infections, and growth during the first two years of life in discordant mother-infant pairs. Data from *n* = 539 mother-infant pairs in the Ulm SPATZ Health Study (SPATZ) and *n* = 488 pairs in the Ulm Birth Cohort Study (UBCS) were analysed using logistic, modified Poisson, and linear regression models. A significant proportion of infants in both cohorts were heterozygous secretors (46% in SPATZ and 45% in UBCS), and about a third had discordant secretor status with their mothers. Non-secretor infants receiving secretor milk in SPATZ showed a significant increase in BMI between week 4/5 and month 3/4 compared to secretor infants. But these associations were not significant after adjusting for confounders and correcting for multiple testing (α threshold = 0.0035). Despite the non-significant results, the findings emphasize the need for further research on discordant secretor status and its health implications.

**Supplementary Information:**

The online version contains supplementary material available at 10.1038/s41598-025-28217-w.

## Introduction

Infants are protected against early infections by their own immunity and a supply of bioactive components through human milk. Mucosal activity plays a crucial role in maintaining immune homeostasis and protecting against pathogens. The fucosyltransferase (FUT2) gene encoded the enzyme α-1,2-fucosyltransferase (Se), which influences this process by determining the presence of specific blood group antigens on mucosal surfaces and in body fluids, including human milk^1^. The activity of the FUT2 gene affects the types of sugars present on epithelial cells, thereby modulating their protective functions and susceptibility to diseases. There are several polymorphisms for the FUT2 gene, of which, secretors have at least one functional FUT2 allele, and non-secretors are homozygous for the inactive FUT2 allele^1^. However, evidence regarding the impact of FUT2 polymorphism on respiratory and ear infections, as well as early childhood growth, remains limited.

On one hand, maternal secretor status influences the abundance of human milk oligosaccharides (HMOs)^2^, which have functional effects in early childhood^3–6^. Secretor and non-secretor milk phenotypes have distinct HMO profiles, based on the presence, abundance and/or absence of specific HMOs^7–10^. Thus breastfeeding from secretor mothers has been shown to be the most effective method of transferring fucosylated HMOs to infants^11–13^. Further, certain commercial formulas have started incorporating HMOs into their products, typically featuring α1,2-fucosylated HMOs, which are not naturally produced by non-secretor mothers^14^. Yet, the impact of providing secretor-type HMOs to non-secretor infants remains clear.

Maternal secretor phenotype i.e. individual HMOs used as proxies for secretor milk have been associated with lower risk of infections^3,15^, and a protective association with atopic dermatitis (AD) has been reported^16^ for infants with high allergy risk. Some studies also reported that children of non-secretor mothers had a higher BMI at 3 and 6 months^17^, although others did not confirm this^18,19^. A maternal FUT2 secretor genotype is associated with lower risk of acute respiratory tract infections in their infants^20^, while a FUT2 non-secretor genotype has been linked to protection against diarrhoea in infants^21^. However, these effects vary regionally; for example, secretor phenotype protects against *C. jejuni* diarrhoea in Mexico^22^, but similar effects could be observed under different conditions in the Philippines^23^. This suggests that the interaction between genetics and environmental factors in diarrheal protection is complex and population-specific.

On the other hand, infants can inherit either at least one functional FUT2 allele (secretors) or two non-functional alleles (non-secretors), which render them non-secretors. The absence of a functional FUT2 gene in non-secretor infants leads to poorly fucosylated mucosal gut surfaces and compromised immune development^24^. For instance, an infant FUT2 secretor genotype has been linked to increased risk for lower respiratory tract infections (LRTI)^25^, and more frequent acute respiratory illnesses^26^., while a non-secretor FUT2 genotype was associated with lower risk of ear infections^27^.

In summary, a FUT2 phenotype or genotype can affect child outcomes in different ways, depending on whether maternal or infant secretor status is considered. Information on both mother and infant secretor status is therefore needed to understand how HMOs and FUT2 polymorphisms influence infant health. However, very few studies^18,28^ have simultaneously considered maternal and infant secretor status. In addition, the role of discordance between maternal and infant FUT2 status^5^, as well as the potential intermediate phenotypes caused by heterozygosity, remain underexplored^1,29^.

### Aims and hypotheses

The aim of this study was to investigate whether the association between maternal secretor status (as defined by milk HMO phenotype) and child health outcomes is modified by the infant’s FUT2 genotype. We specifically focused on otitis media (OM), LRTI, upper respiratory tract infections (URTI) as primary outcomes and AD and child growth as secondary outcomes, in mother-infant pairs with discordant secretor status from the two Ulm Birth Cohort Studies.

We hypothesised that:

i) maternal secretor milk is associated with lower susceptibility to infections and favourable growth outcomes
ii) these associations differ according to the infant’s FUT2 genotype, and
iii) discordance between maternal and infant secretor status further modifies these associations.

## Methods

### Study design and population

Data used in the current study were from two methodologically similar birth cohort studies, the Ulm SPATZ Health Study (SPATZ) and the Ulm Birth Cohort Study (UBCS), which enrolled live new-borns and their mothers from the general population shortly after delivery at the University Medical Centre, in Ulm, Southern Germany. The recruitment periods were from November 2000 to November 2001 for UBCS and from April 2012 to May 2013 for SPATZ^30^. Exclusion criteria were outpatient delivery, maternal age below 18 years, immediate transfer of the newborn or mother to intensive care after delivery, and/or insufficient proficiency in German (for both UBCS and SPATZ) or Turkish or Russian (for UBCS only) languages. At baseline, UBCS included 1090 live newborns from 1066 mothers (67% of all 1593 eligible families), while SPATZ included 1006 live newborns from 970 mothers (49% of all 1999 eligible families). For this analysis, data were restricted to singleton births. Ethical approval was obtained from the ethics board of Ulm University (UBCS: No. 98/2000; SPATZ: No. 311/11) and from the Physicians’ Boards of the states of Baden-Württemberg and Bavaria (for UBCS only). All research procedures and methods were conducted in accordance with the Declaration of Helsinki. Participation was voluntary, and written informed consent was obtained from each participant. Definitions of exposure, outcome, and confounders, as well as statistical methods, were consistent between both studies unless specifically stated otherwise.

### Data collection and measurements

Demographic information, maternal and birth-related data (including child sex, maternal age, pre-pregnancy weight, and body mass index (BMI)), and maternal and infant history of allergic disease were gathered through self-administered questionnaires, hospital records, and routine screening during pregnancy. Maternal allergy status was determined through self-reported history of physician-diagnosed hay fever, neurodermatitis, or asthma, with non-allergic mothers identified as those reporting no history of allergic disease. Infant weight and length measurements were obtained during routine paediatric appointments at 3–10 days, 4–5 weeks, 3–4 months, 6–7 months, 10–12 months, and 21–24 months of age. For this study, assessments conducted at 4–5 weeks, 3–4 months, 6–7 months and 10–12 months were used. In UBCS, follow-ups were done using self-administered questionnaires at ages 1, 2, 3, 4, 6, 8, 11, 13, 15, and 17 years. Yearly follow-ups are still ongoing in SPATZ.

### FUT2 genotyping

The child’s FUT2 genotype was determined using cord blood. DNA was extracted manually using the FlexiGene DNA Kit (Quiagen), genotyping was performed with the Infinium Global Screening Array-24 + V1.0 GSA + Multi Disease (Illumina), and genome-wide data were imputed using the imputation reference of the 1000 Genomes Phase 3.

Infant FUT2 secretor status was defined using three genotyped SNPs, namely rs516246, rs602662, rs281379, [Minor Allele Frequency (MAF): SPATZ = 0.195, UBCS = 0.171] with genotypes classified as homozygous wild type, heterozygous, or homozygous mutant. In one step, like previous studies^29,31–34^, infants were grouped into three categories: secretors (homozygous wild type and/or heterozygous for all SNPs), non-secretors (homozygous mutant for all SNPs), and those with a mixed secretor status (a combination of homozygous wild type/heterozygous and homozygous mutant for any SNP). In a second step, homozygous wild type secretors heterozygous secretors, and non-secretors each had the same genotype for all three SNPs. The mixed status group had a combination of genotypes, indicating both secretor and non-secretor status across the SNPs, which could potentially confer intermediate susceptibility to disease in comparison to full secretor or non-secretor infants.

In SPATZ, maternal secretor status, milk phenotypes and milk group were based on the presence or absence of α1,2- and α1,4-fucosylated human milk oligosaccharides (HMOs) measured at 6 weeks of lactation as previously described^8^. In UBCS, Lewis A and Lewis B antigens were measured in maternal serum collected immediately after delivery as described elsewhere^35^. Maternal secretor status, used as a proxy for milk phenotype, was based on Lewis blood group antigens: Le (A-B+) and Le (A + B+) were classified as secretors, while Le (A + B-) and Le (A-B-) were non-secretors.

### Outcome assessment and definitions

Atopic dermatitis at 2 years:

Atopic dermatitis (AD) diagnosed by a doctor was assessed by parent and paediatrician reports using separate questionnaires at 1 and 2 years of age. Parent-reported AD diagnosis was indicated by a positive response to the question “Has a doctor diagnosed your child with neurodermatitis (in SPATZ; endogenous eczema, atopic dermatitis) or atopic dermatitis (in UBCS) in the past 12 months?” Paediatrician-reported AD diagnosis was determined by a positive response to the question “Has a doctor diagnosed your child with neurodermatitis (endogenous eczema, atopic dermatitis) until now?” Although labelled as “parent-reported” and “paediatrician-reported” AD, both reports indicated a doctor’s diagnosis, although not necessarily made by the responding paediatricians themselves. For the purpose of this analysis, children with a positive AD diagnosis reported by either parents or paediatricians at 1 and/or 2 years were classified as AD cases.

Infections at 2 years:

In both studies, reported doctor’s diagnoses of OM, LRTI (including pneumonia, bronchitis, pertussis, tracheobronchitis, Krupp, bronchiolitis and flu), and upper respiratory tract infection (URTI; including rhinitis, pharyngitis, tonsillitis and epiglottitis) in the first and second years of life, were assessed using standardised, self-administered questionnaires from the children’s primary care paediatricians. Several other health outcomes were also similarly assessed concurrently using these questionnaires. Children with a positive report of OM, LRTI or URTI at 1 and/or 2 years of age were considered OM, LRTI or URTI cases, respectively.

### Growth (BMI)

**Fig. 1:**
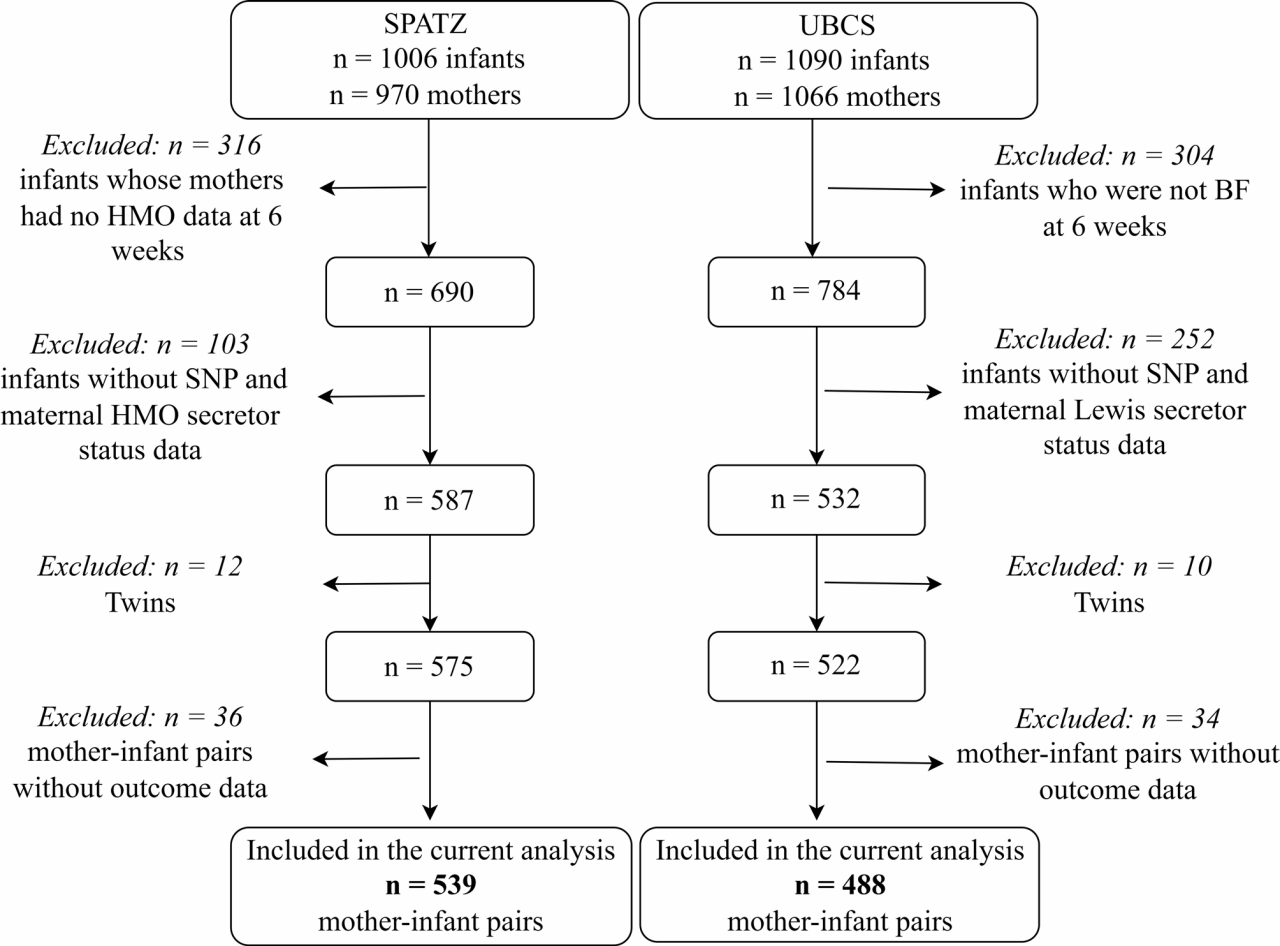
Flow diagram of mother-child pairs included the current analysis. SPATZ: Ulm SPATZ Health Study; UBCS: Ulm Birth Cohort Study; HMO: human milk oligosaccharides, SNP: single nucleotide polymorphisms; BF: breastfeeding.

Child BMI was calculated using standard anthropometric measurements (kg/m^2^) and subsequently transformed into z-scores adjusted for precise age at measurement (in days) and the child’s sex as previously described^36^. Changes in BMI were assessed as percentage change between periods, which were then converted to z-scores, adjusting for the child’s gender and the duration (in days) between measurements. To ensure that associations reflected those in the source populations, all z-scores were derived relative to their respective study populations (UBCS or SPATZ) rather than from standardized growth charts^37^. A total of *n* = 539 and *n* = 488 mother-infant pairs were included in the current analysis (Fig. 1). To ensure direct comparisons of exposure to secretor or non-secretor milk, the UBCS study samples were further restricted to infants who were breastfeeding at 6 weeks, which aligns with the time of sample collection for HMO assessment in SPATZ.

### Statistical analyses

Demographic characteristics were summarized with descriptive statistics. Associations between secretor and non-secretor milk with infant secretor status (secretor, non-secretor, or mixed) and outcomes (AD, OM, LRTI, URTI, BMI) were assessed using three models. Model 1 explored the association across infant secretor status categories receiving secretor or non-secretor milk. Model 2 examined the potential impact of FUT2 heterozygosity by comparing the four genotypes (homozygous wild type, heterozygous, homozygous mutant, mixed status) of infants receiving secretor or non-secretor milk. Model 3 focused on the maternal secretor vs. non-secretor milk phenotype among non-secretor infants. To assess discordance, concordant infants were used as reference groups (e.g., secretor infants in the secretor milk group, and non-secretor infants in the non-secretor milk group). Thus, associations were investigated as follows: secretor milk to non-secretor and/or mixed status infants, and non-secretor milk to secretor and/or mixed status infants. The odds ratio (OR) for AD, relative risk (RR) for infections, β estimate for growth and 95% confidence intervals (95% CI) were calculated using logistic, modified Poisson, and general linear regression models, respectively, in the three afore-mentioned models, crude and adjusted. Adjusted models included relevant factors like maternal allergy, child sex, delivery mode, birth weight, and gestational age, selected depending on the outcome. This selection was guided by a priori knowledge from the literature, which has established these factors as potential confounders in associations involving maternal secretor status (or related HMOs), infant genotype and child health outcomes due to their influence on immune development, microbial colonisation and early growth. This includes studies on secretor status and allergy risk^29^, delivery mode effects on gut microbiota^38,39^, impact of gestational age/birthweight^40^ and sex differences^41^ on paediatric outcomes. To confirm their relevance in our study populations, we additionally assessed the differences in the distribution of child sex, delivery mode, gestation age, birthweight, and pre-pregnancy BMI between maternal and child secretor status, which were tested using Chi-square and Kruskal-Wallis tests. Differences in the distribution of gestational age and birthweight were statistically significant in SPATZ only, while differences in child sex, and delivery mode were statistically significant in UBCS only. A Bonferroni-adjusted threshold was used as a level of statistical significance following correction for multiple testing, with an α threshold = 0.0035 accounting for 14 significance tests. A pooled analysis combining data from the two cohorts was conducted as part of a sensitivity analysis to evaluate the robustness of the results. Statistical models for the pooled analyses included an indicator variable for cohort to adjust for potential baseline differences between the studies. This analysis tested whether associations observed in the individual datasets remained consistent when the cohorts were combined. All statistical analyses were performed in SAS (version 9.4, Carey, NC, US) and R (version 3.5.1; R Foundation for Statistical Computing).

## Results

### Characteristics of study sample

Data from a total of *n* = 539 and *n* = 488 mother-infant pairs in SPATZ and UBCS, respectively, were used in the current analysis (Table 1). Almost half of the infants were heterozygous secretors (46% in SPATZ, and 45% in UBCS); less than 20% were non-secretors (i.e. homozygous mutant; 17% in SPATZ and 15% in UBCS). The majority of infants (80%) in SPATZ and more than two thirds (71%) in UBCS had secretor mothers, thus exposure to secretor milk. Discordant secretor status pairs comprised almost a third (27%) in SPATZ and more than a third (33%) in UBCS. Characteristics stratified according to mother-child secretor status matches are shown in Supplementary **Tables S1-S2**. Detailed sample sizes for each subgroup included in the following stratified analyses are presented in Supplementary **Tables S3** and **S4**.

**Table 1:** Demographic characteristics of mother-infant pairs in the Ulm birth cohort studies.

	SPATZ (*n* = 539)			UBCS (*n* = 488)		
	*n*	% or mean	(95%LCL; 95%UCL)	*n*	% or mean	(95%LCL; 95%UCL)
Child sex						
Male	287	53	(49.0; 57.5)	243	49.8	(45.4; 54.2)
Female	252	47	(42.5; 51.0)	245	50.2	(45.8; 54.6)
Birth weight (g)	538	3390.8	(3352.0; 3429.7)	488	3405.4	(3364.8; 3446.0)
Exclusive breastfeeding						
Yes	425	79	(75.4; 82.3)	478	98	(96.7; 99.2)
No	114	21	(17.7; 24.6)	10	2	(0.8; 3.3)
FUT2 heterozygousity^1^						
Homozygous wild type	143	27	(22.8; 30.3)	150	31	(26.6; 34.8)
Heterozygous	246	46	(41.4; 49.8)	218	45	(40.3; 49.1)
Homozygous mutant	92	17	(13.9; 20.2)	74	15	(12.0; 18.3)
Mixed status	58	11	(8.1; 13.4)	46	9	(6.8; 12.0)
Secretor status^2^						
Secretor	421	78	(74.6; 81.6)	395	81	(77.5; 84.4)
Non-secretor	92	17	(13.9; 20.2)	74	15	(12.0; 18.3)
Both	26	5	(3.0; 6.6)	19	4	(2.2; 5.6)
AD up to 2 years						
Yes	106	25	(20.8; 29.0)	110	34	(28.8; 39.1)
No	320	75	(71.0; 79.2)	214	66	(60.9; 71.2)
OM up to 2 years						
Yes	120	28	(23.4; 31.8)	177	49	(43.5; 53.8)
No	315	72	(68.2; 76.6)	187	51	(46.2; 56.5)
LRTI up to 2 years						
Yes	207	45	(40.2; 49.2)	319	77	(73.4; 81.5)
No	256	55	(50.8; 59.8)	93	23	(18.5; 26.6)
URTI up to 2 years						
Yes	372	81	(77.3; 84.5)	168	39	(34.5; 43.8)
No	88	19	(15.5; 22.7)	261	61	(56.2; 65.5)
Maternal age (years)	538	33.2	(32.8; 33.6)	487	32.1	(31.7; 32.5)
Parity (n births of foetus ≥ 24 weeks)						
0 births	280	52	(47.8; 56.3)	235	48	(43.7; 52.6)
≥ 1 birth	258	48	(43.7; 52.2)	253	52	(47.4; 56.3)
Gestational age at delivery (weeks)						
≤ 36	30	6	(3.6; 7.5)	18	4	(2.0; 5.4)
Between 36 and 41	423	79	(75.2; 82.1)	367	76	(71.9; 79.5)
≥ 41	85	16	(12.7; 18.9)	100	21	(17.0; 24.2)
Delivery mode^3^						
Vaginal	428	80	(76.1; 83.0)	414	85	(81.7; 88.0)
Cesarean	110	20	(17.0; 23.9)	74	15	(12.0; 18.3)
Maternal secretor status^4^						
Yes	431	80	(76.6; 83.3)	344	71	(66.4; 74.5)
No	108	20	(16.7; 23.4)	144	30	(25.5; 33.6)
Maternal ABO blood group						
A	224	43	(38.9; 47.4)	198	41	(36.5; 45.2)
B	74	14	(11.3; 17.3)	58	12	(9.1; 14.8)
O	199	38	(34.2; 42.5)	211	44	(39.1; 47.9)
AB	22	4	(2.5; 6.0)	18	4	(2.0; 5.4)
Rhesus factor						
Positive	426	82	(79.1; 85.7)	411	85	(81.5; 87.9)
Negative	91	18	(14.3; 20.9)	74	15	(12.1; 18.5)
Maternal milk group/Lewis blood group (UBCS)^5^						
I/A−B+	399	74	(70.3; 77.7)	341	70	(65.8; 73.9)
II/A + B−	101	19	(15.4; 22.0)	77	16	(12.5; 19.0)
III/A−B−	32	6	(3.9; 7.9)	67	14	(10.7; 16.8)
IV/A + B+	7	1	(0.3; 2.3)	3	1	(0.0; 1.3)
Mother-child secretor matches						
Secretor to secretor	359	67	(62.6; 70.6)	293	60	(55.7; 64.4)
Non-secretor to non-secretor	40	7	(5.2; 9.6)	35	7	(4.9; 9.5)
Secretor to non-secretor	52	10	(7.2; 12.1)	39	8	(5.6; 10.4)
Non-secretor to secretor	62	12	(8.8; 14.2)	102	21	(17.3; 24.5)
Status mix	26	5	(3.0; 6.6)	19	4	(2.2; 5.6)

AD, Atopic dermatitis; OM: Otitis media, LRTI: Lower respiratory tract infections; URTI, Upper respiratory tract infections; SPATZ, Ulm SPATZ Health Study; UBCS, Ulm Birth Cohort Study. Sums (*n*) may not always add up to the total because of missing values for certain items.

^1^Infant fucosyltransferase-2 (FUT2) secretor status was determined from the three single-nucleotide polymorphisms (SNPs), rs281379, rs516246 and rs602662.

^2^*Secretors*: SeSe and/or Sese for all SNPs; *non-secretors*: sese for all SNPs; *Mixed*: combination of homozygous mutant and other genotypes.

^3^Delivery mode: spontaneous or assisted vaginal delivery; or elective/emergency caesarean section.

^4^Maternal secretor status based on human milk oligosaccharides (HMOs) in SPATZ or Lewis blood group antigens in UBCS. Secretors: A-B + or A + B+, non-secretors: A + B− or A−B−.

^5^Maternal milk group was defined by HMO quantification in SPATZ, or on Lewis AB status in UBCS, shown in parentheses.

### Associations of child secretor status with health outcomes

**Fig. 2:**
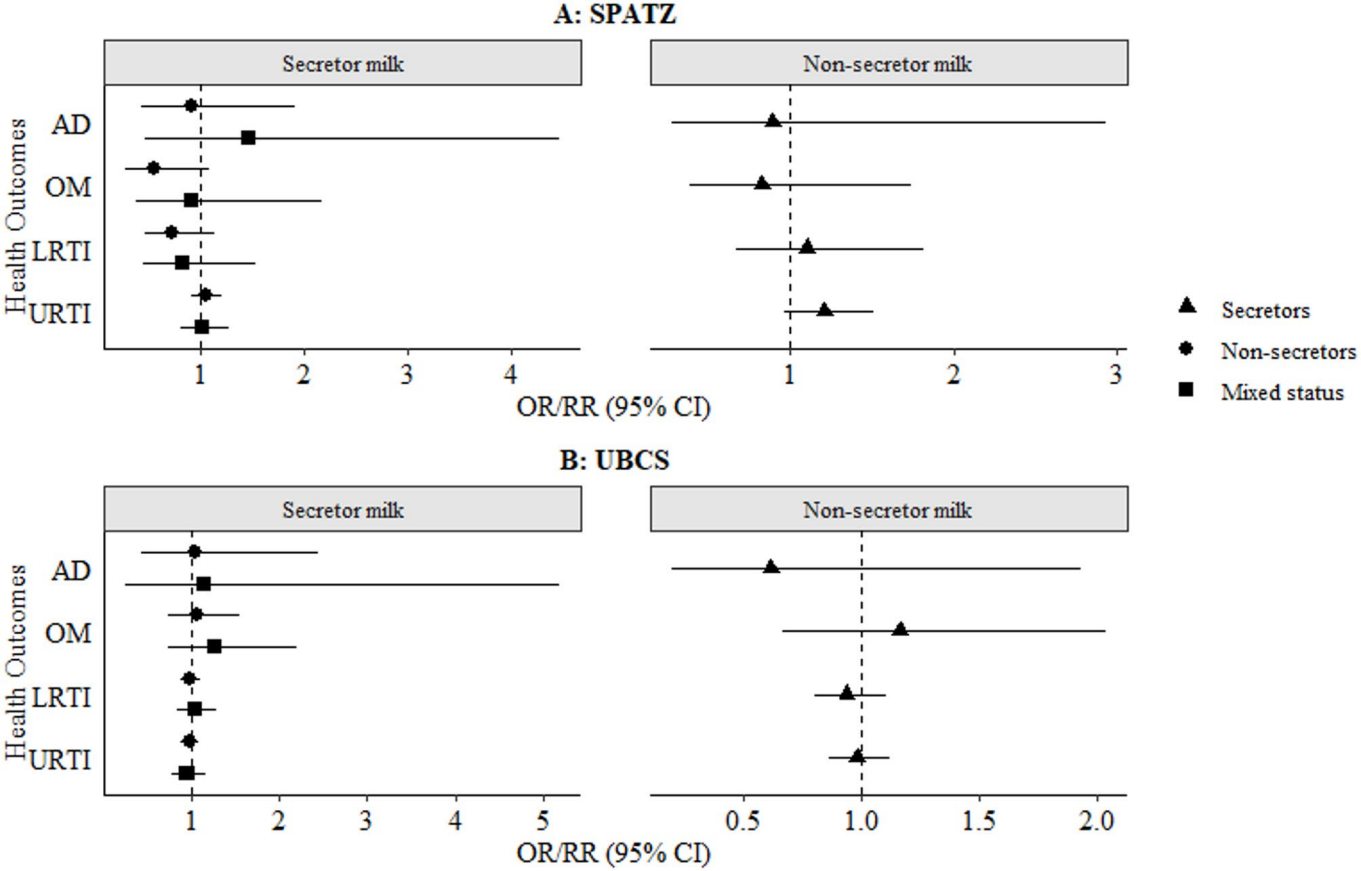
Adjusted associations between infant fucosyltransferase-2 (FUT2) secretor status and likelihood of developing atopic dermatitis (AD), lower respiratory tract infections (LRTI) and upper respiratory tract infections (URTI) up to 2 years of age, stratified by maternal secretor and non-secretor milk phenotypes. Results are shown as odds ratios (AD) and risk ratios (infections) with 95% confidence intervals (CI). Models were adjusted for relevant covariates (child sex, maternal allergy for AD, delivery mode for infections, maternal milk and/or ABO blood group). Bonferroni-adjusted significance threshold: α = 0.0035. AD, atopic dermatitis; OM, otitis media; LRTI, lower respiratory tract infections; URTI, upper respiratory tract infections; CI, confidence interval; FUT2, fucosyltransferase-2; SNP, single nucleotide polymorphism; HMO, human milk oligosaccharide; SPATZ, Ulm SPATZ Health Study; UBCS, Ulm Birth Cohort Study.

The associations of child secretor status with AD and infections at 2 years were assessed in crude and adjusted models. There were no statistically significant (α threshold = 0.0035) associations [odds ratio (OR)/risk ratio (RR) (95% confidence interval (CI))] between child secretor status and AD, OM, LRTI and URTI, amongst children receiving secretor or non-secretor milk (Fig. 2, Supplementary **Figure S1**).

### Associations of child secretor status with change in BMI

**Fig. 3:**
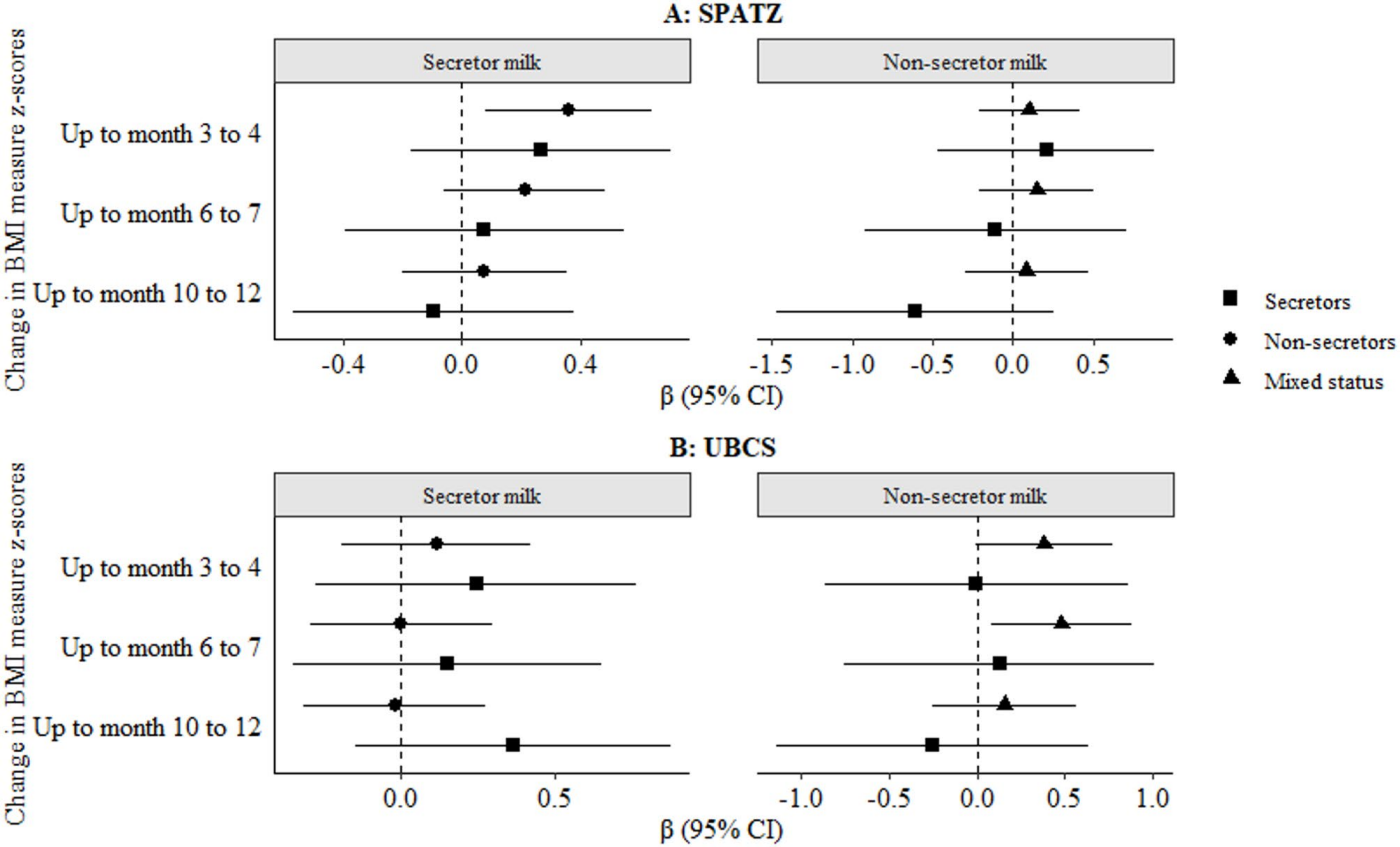
Adjusted associations between infant fucosyltransferase-2 (FUT2) secretor status and change in child body mass index (BMI) z-scores in the first year of life stratified by secretor and non-secretor milk phenotypes. Estimates (β, 95% CI) were obtained from general linear models adjusted for sex, age, maternal milk and/or ABO group, birthweight, and gestational age. Bonferroni-adjusted significance threshold: α = 0.0035. BMI, body mass index; CI, confidence interval; FUT2, fucosyltransferase-2; SNP, single nucleotide polymorphism; HMO, human milk oligosaccharide; SPATZ, Ulm SPATZ Health Study; UBCS, Ulm Birth Cohort Study.

We also investigated the associations of child secretor status with change in BMI from the 4 to 5 week measure, up to 3 to 4 months, 6 to 7 months, and 10 to 12 months, amongst children receiving secretor or non-secretor milk at 6 weeks in crude (Supplementary **Figure S2**) and adjusted models (Fig. 3). In SPATZ crude models, non-secretor infants receiving secretor milk showed a statistically significant (α threshold = 0.0035) increase in BMI from week 4/5 to month 3/4 [β (95%CI): 0.45 (0.17–0.73), *p* = 0.0018, Supplementary **Figure S2A**]. In the adjusted model, this association was only nominally significant [β (95%CI): 0.36 (0.08–0.64), *p* = 0.01, Fig. 3A].

Again, only nominally significant, a similar association was observed in UBCS [β (95%CI): 0.30 (0.02–0.57), *p* = 0.032, Supplementary **Figure S2B**], but with no statistical significance in adjusted models (Fig. 3B). However, secretor infants of non-secretor mothers, thus receiving non-secretor milk, also had a positive change in BMI up to month 6/7 [β (95%CI): 0.48 (0.08–0.89), *p* = 0.01, Fig. 3A). These and other associations were not statistically significant following adjustment for multiple testing (α threshold = 0.0035), but showed the same direction in both studies.

### Associations between child FUT2 heterozygosity and health outcomes

**Fig. 4:**
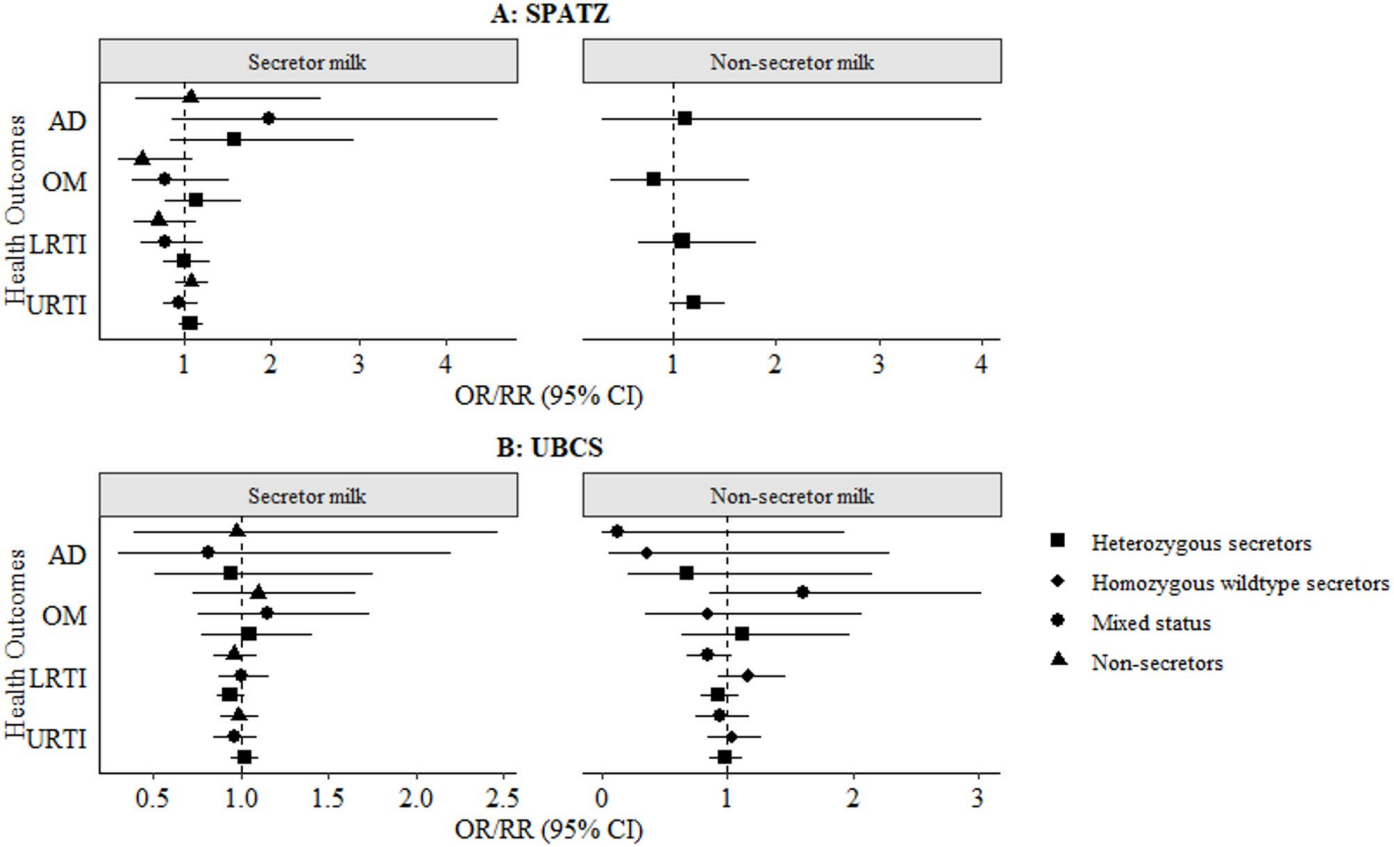
Adjusted associations between infant fucosyltransferase-2 (FUT2) heterozygosity with health outcomes in the first 2 years of life, stratified by secretor and non-secretor milk in the Ulm Birth Cohorts. Results are presented as odds ratios (AD) and risk ratios (infections) with 95% CI. Models adjusted for child sex, maternal allergy (AD only), delivery mode (infections only), maternal milk and/or ABO blood group. Reference groups: homozygous secretors (SeSe) in secretor milk and non-secretors (sese) in non-secretor milk. Bonferroni-adjusted significance threshold: α = 0.0035. Bonferroni-adjusted level of statistical significance is α = 0.05/14 = 0.0035. Abbreviations: AD, atopic dermatitis; OM, otitis media; LRTI, lower respiratory tract infections; URTI, upper respiratory tract infections; CI, confidence interval; FUT2, fucosyltransferase-2; SNP, single nucleotide polymorphism; HMO, human milk oligosaccharide; SPATZ, Ulm SPATZ Health Study; UBCS, Ulm Birth Cohort Study.

In a second step, we investigated the associations between child FUT2 heterozygosity and health outcomes i.e. AD, OM, LRTI and URTI. There were no statistically significant associations between child FUT2 heterozygosity and health outcomes at 2 years amongst infants receiving secretor or non-secretor milk, in both crude (Supplementary **Figure S3**) and adjusted models (Fig. 4), at neither conventional level of significance (*p* < 0.05) nor following correction for multiple testing (α threshold = 0.0035).

### Associations between child FUT2 heterozygosity and BMI outcomes

**Fig. 5:**
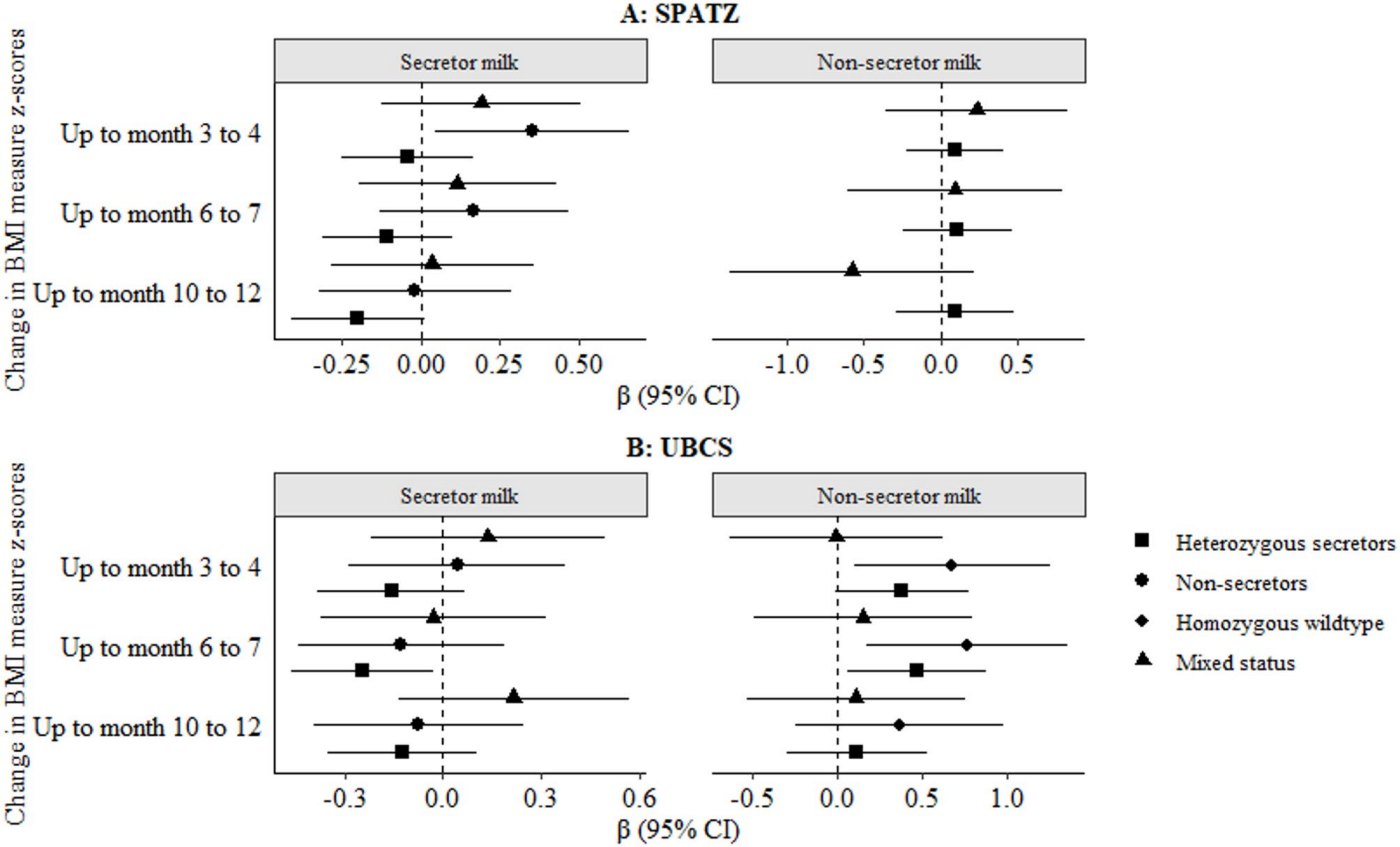
Adjusted associations between infant fucosyltransferase-2 (FUT2) heterozygosity and change in infant body mass index (BMI) in the first year of life stratified by secretor and non-secretor milk in the Ulm Birth Cohorts. Results (β, 95% CI) from general linear models adjusted for sex, age, maternal milk and/or ABO blood group, birthweight, and gestational age. Change in BMI defined as difference between BMI at 4–5 weeks and subsequent time points. Bonferroni-adjusted significance threshold: α = 0.0035. Abbreviations: BMI, body mass index; CI, confidence interval; FUT2, fucosyltransferase-2; SNP, single nucleotide polymorphism; HMO, human milk oligosaccharide; SPATZ, Ulm SPATZ Health Study; UBCS, Ulm Birth Cohort Study.

We also investigated the impact of child FUT2 heterozygosity, on the change in BMI in the first year of life, amongst infants receiving secretor or non-secretor milk. There were no statistically significant associations following correction for multiple testing in both crude and adjusted models (Fig. 5, Supplementary **Figure S4**). We did observe some potential associations that were statistically significant at conventional level (*p* < 0.05). For example, non-secretor infants, receiving secretor milk had a positive change in BMI from the week 4 to 5 measure up to month 3 to 4 in SPATZ crude models [β 95% CI: 0.45 (0.15–0.76), *p* = 0.0039; Supplementary **Figure S4A**]. Directionality was the same in UBCS, but this association was not statistically significant (Supplementary **Figure S4B**). Likewise, heterozygous secretor infants receiving secretor milk in UBCS, had a negative change in BMI from the week 4 to 5 measurement up to the month 6 to 7 measurement [β 95% CI: -0.25 (-0.48 – -0.02), *p* = 0.034; Supplementary **Figure S4B**].

In adjusted models, there were no statically significant associations in SPATZ (Fig. 5A), and only the association in UBCS was statistically significant again at conventional level i.e. *p* < 0.05, [β 95% CI: -0.25 (-0.46 – -0.03), *p* = 0.025; Fig. 5B]. Additionally, heterozygous secretor infants receiving non-secretor milk in UBCS, had a positive change in BMI from the week 4 to 5 measure up to month 6 to 7 measure [β 95% CI: 0.47 (0.06–0.88), *p* = 0.02]. While, homozygous wild type secretors receiving non-secretor milk had a positive change in BMI up to the 3 to 4 months [β 95% CI: 0.67 (0.09–1.25, *p* = 0.02] and 6 to 7 months measure [β 95% CI: 0.76 (0.17–1.35, *p* = 0.01]. Still, all these were not statistically significant following correction for multiple testing (α threshold = 0.0035), but directionality was similar in SPATZ (Fig. 5).

### Associations of milk phenotype with health and BMI outcomes amongst non-secretor infants

**Fig. 6:**
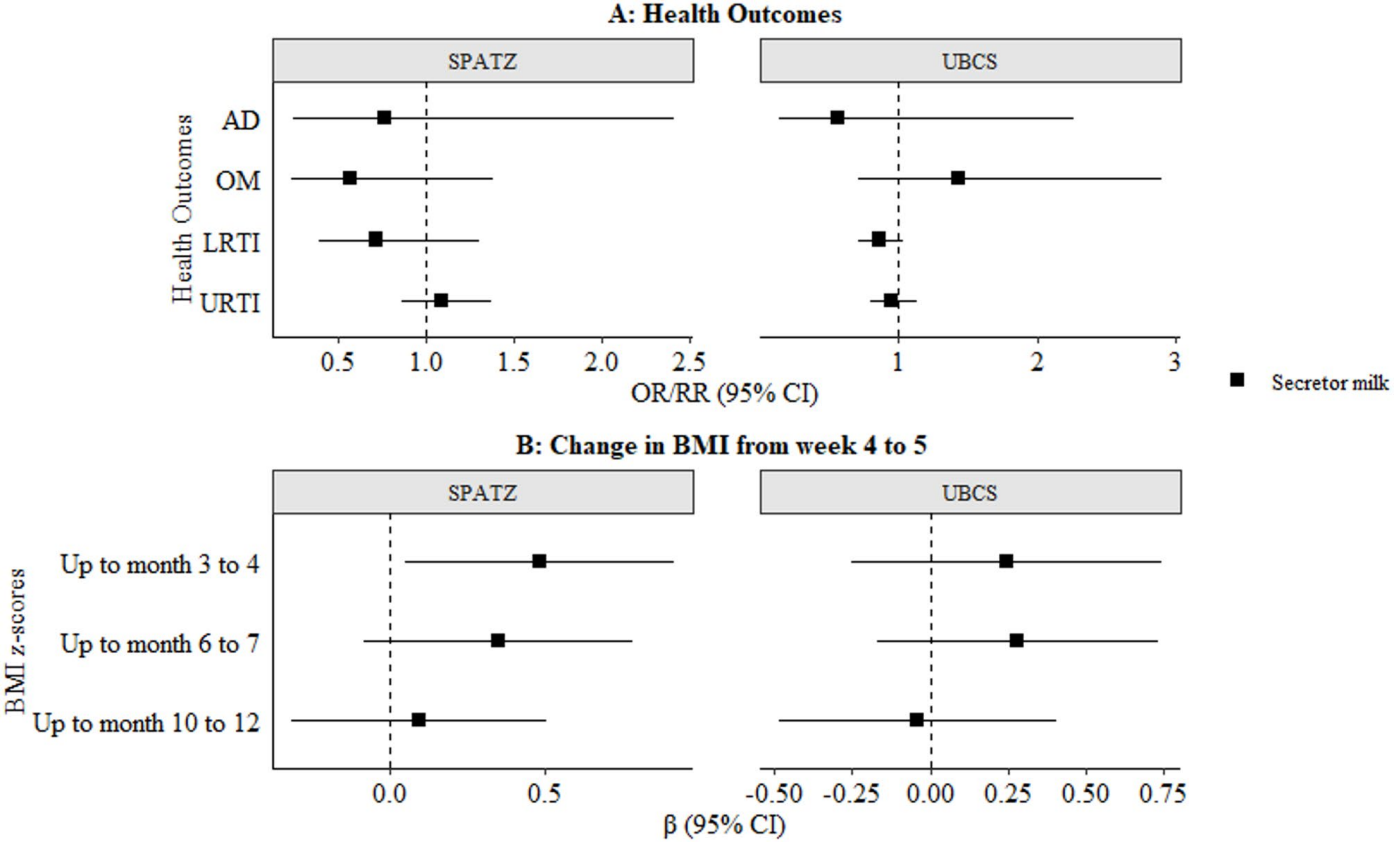
Adjusted associations maternal secretor status with (**A**) likelihood of infections in the second year of life and (**B**) change in child body mass index (BMI) amongst non-secretor infants. Results are shown as odds ratios (AD), risk ratios (infections), and β estimates (BMI) with 95% CI. Models adjusted for child sex, maternal allergy (AD only), delivery mode (infections only), maternal milk group and/or ABO blood group, birthweight, and gestational age. Bonferroni-adjusted significance threshold: α = 0.0035. Abbreviations: AD, atopic dermatitis; OM, otitis media; LRTI, lower respiratory tract infections; URTI, upper respiratory tract infections; BMI, body mass index; CI, confidence interval; FUT2, fucosyltransferase-2; SNP, single nucleotide polymorphism; HMO, human milk oligosaccharide; SPATZ, Ulm SPATZ Health Study; UBCS, Ulm Birth Cohort Study.

We further investigated the association of secretor milk vs. non-secretor milk with health outcomes and change in BMI amongst non-secretor infants (Fig. 6, Supplementary **Figure S5**). There were no statistically significant associations with health outcomes, at conventional level or following correction for multiple testing (α threshold = 0.0035) **(**Fig. 6A). In SPATZ, secretor milk was associated with a positive change in BMI up to the 3 to 4 months measure [β 95%CI: 0.60 (0.19–1.02), *p* = 0.0044], and 6 to 7 month measure [β 95%CI: 0.49 (0.08–0.90), *p* = 0.019, Supplementary **Figure S5B**] in crude models. But, only up to the 3-to-4-month measurement in adjusted models [β 95%CI: 0.48 (0.05–0.92), *p* = 0.03, Fig. 6B). However, after adjustment for multiple testing (α threshold = 0.0035), there were no statistically significant associations in SPATZ, although directionality was similar in UBCS (Fig. 6). Similar results were also observed in the pooled analysis (Supplementary Tables S5-S8).

## Discussion

The current study investigated associations between child FUT2 secretor status and health outcomes up to 2 years as well as change in BMI in the first year of life, amongst children receiving secretor or non-secretor milk in the Ulm Birth Cohorts. Less than 20% of the infants were genetic non-secretors; almost 50% were heterozygous secretors. A third of the mother-infant pairs had discordant secretor status in both studies. In crude models, non-secretor infants had a statistically significant positive change in BMI from the week 4 to 5 measurement up to the 3 to 4 months measurement. In addition, we observed some associations that were also statistically significant at nominal level (*p* < 0.05). However, all these associations were not statistically significant following adjustment for potential confounding factors and correction for multiple testing (α threshold = 0.0035).

Firstly, the concept of maternal-infant pairs based on discordant secretor status as presented in this study, represents a novel frontier in paediatric research^5^, and provides insight into the intricate dynamics of mother-infant interactions and their implications on health outcomes. In addition, the distribution of infants with a non-secretor genotype in our study was in line with expected low prevalence in other populations^1,42^. Although our findings were not statistically significant, our results do show a potential association of FUT2 heterozygosity on child outcomes which is in line with other reports^1,43^. We do show that potential variations in secretor status between mothers and infants could exert independent effects on child outcomes. Thus, the potential significance of discordant secretor status could help uncover novel insights into the complex interplay between genetic predisposition and nutritional exposure with susceptibility to disease and differences in growth during early childhood.

Of note, discordant maternal-infant secretor status may influence early life outcomes through several biological mechanisms. Secretor milk is rich in α1,2-fucosylated HMOs which are selectively involved in beneficial bacteria and can act as decoys for pathogens^44^. As such, infants whose genotype does not match the maternal milk HMO profile may experience altered microbial colonisation, nutrient utilisation or immune modulation, which could potentially affect BMI trajectories and infection susceptibility. However, given the small percentage of non-secretors, future studies should ensure the enrolment of an adequate sample size of non-secretor mothers. This would facilitate thorough analyses and aid in providing tailored recommendations for the underrepresented demographic population^5^.

Secondly, there were no statistically significant associations between infant FUT2 genotypes with AD, and/or infections in the first 2 years or life, among infants receiving secretor or non-secretor milk. These findings are also in line with a large cohort study conducted in the United Kingdom analysing *n* = 1831 mother-child pairs^25^, which did not find any statistically significant associations between infant FUT2 secretor genotype, with ear infections, atopic eczema or atopy. But, the authors in that study^25^ reported that non-secretor infants had an increased susceptibility to lower respiratory illness (pneumonia or bronchiolitis) between 12 and 24 months, but not in early infancy. Also, maternal FUT2 genotype did not influence infant health outcomes, when considering infant FUT2 genotype. On one hand, infants of secretor mothers had a significantly lower incidence of acute respiratory illness (ARI) during the first 6 months of life in another study^20^. On the other hand, a post hoc analysis revealed an increased risk of ARIs for secretor infants only, with and without accounting for the maternal secretor status^20^. An earlier study also found that secretors across all age groups formed a larger proportion of cases with respiratory infection and/or a diagnosis or respiratory viruses^45^. Despite our null results, it is noteworthy to mention, that none of the afore-mentioned studies explored these associations in pairs with discordant secretor statuses, which may mask subtle gene-nutrient or gene-microbiome interactions that influence susceptibility. Thus, further research is needed to elucidate the interplay between discordant maternal-infant secretor status and infections or allergic disease in early childhood.

Furthermore, we found that in crude models, non-secretor infants receiving secretor milk had a significantly increased BMI during the first 4 months of life, compared to non-secretor infants receiving non-secretor milk. Nonetheless, these associations were not statistically significant in adjusted models. Several studies have investigated associations between individual HMOs and growth^5^, but only two^17,19^ have compared secretor and non-secretor milk, and one other study^18^ compared secretor-positive and secretor negative milk, all with conflicting results. For instance, two studies^18,19^ found no associations while the other study^17^ reported that infants receiving non-secretor milk had a significantly higher BMI at 3 and 6 months. In contrast to these findings, we found that secretor infants of non-secretor mothers, thus receiving non-secretor milk had higher BMI at 6 to 7 months, in UBCS, although also not statistically significant (Supplementary Figure S6 – S7).

We further found that in UBCS, heterozygous secretors receiving secretor milk had a statistically significant (α threshold = 0.0031) lower BMI at the 6 to 7-month measure in crude models, and at conventional level of significance (*p* < 0.05) in adjusted models (Supplementary Figure S8-S9). This association was only significant at conventional level (*p* < 0.05) in pooled models (Supplementary Table S7). Additionally, there were no statistically significant associations between secretor milk and BMI among non-secretor infants (Supplementary Figure S10). Still, all this indeed highlights the need to consider the secretor status of both the infant and the mother when looking at overall growth outcomes^5^. Yet, only one study^18^ considered infant secretor status, and also reported that maternal secretor status and the respective HMO variation do not seem to play a role in infant growth in the first four months. Of note, the sample sizes of infants receiving non-secretor milk in these studies were also smaller (*n* = 21, *n* = 11, and *n* = 16, respectively) compared to our present study. Moreover, these studies^17–19^ focused on cross-sectional measurements which may not be useful to identify clinically meaningful trajectory patterns for both growth and adverse health outcomes^46^. Given the research gap on the role that an infant’s secretor status plays in modulating outcomes in early childhood, particularly in discordant mother-infant pairs, further research is needed to provide deeper mechanistic insights into how HMO exposure and infant genetics interact to shape growth and immune development. The observations on FUT2 may also warrant further stratification of the four milk types in a broader sense. Secretors produce human milk that is high in α1,2-fucosylated oligosaccharides, whereas milk produced by non-secretors lacks these types of HMOs or only contains minute levels. Previous studies typically defined secretor milk phenotype based on the presence of 2’-FL, an α1,2-fucosylated HMO. Considering this, the identification of 2’-FL in human milk has become feasible and achievable through point-of-care testing^47^. Be that as it may, we previously showed^8,48^ that a single HMO profiling scheme of a single human milk sample obtained as a single lactation stage may not be sufficient to predict the secretor status of an individual. Thus, there may be cases where additional HMOs are required, or other genetic information is needed.

Strengths of the study include the stratification of our results by secretor status, because of the known differences of HMO composition with respect to secretor status. Despite the small sample size of non-secretors, and mismatches, we show associations that may not be present when combining secretors and non-secretors. In addition, we could also distinguish between heterozygous and homozygous secretors, given the differences that may exist between these two genotypes. Furthermore, previous studies^1,32,49^ have highlighted the importance of considering multiple SNPs due to variations in allele frequencies and linkage disequilibrium across diverse populations. As such, the use of three SNPs in our study is a comprehensive approach that enhanced accuracy and robustness in determining infant secretor status.

A major limitation of this study is that the secretor phenotype predominated this cohort, hence a low prevalence of non-secretors, compared to the global level. This resulted in a very small sample size of child outcome cases in the discordant secretor status mother-infant pairs. On one hand, ORs and β estimates for the lower prevalence phenotypes in our study setting may be less precise. These results should therefore be interpreted with caution. Still, we chose to present these analyses for completeness and transparency, as they provide valuable insights into potential patterns across subgroups. On the other hand, the pooled analysis led to a larger sample size numbers and showed similar estimates and results (Supplementary Tables S5-S8). Thus, our results highlight the associations that may not be present when investigating typical secretor-to-secretor or non-secretor-to-non-secretor matches. In addition, maternal secretor milk phenotype was determined differently between SPATZ and UBCS. While secretor status in SPATZ was based on fucosylated HMOs, we used serologically detected Lewis blood group antigens in UBCS. Both methods reflect FUT2-mediated secretor activity and there is evidence of correlations between the serologically detected Lewis blood group and HMOs expressed in human milk^50^. Still, this methodological difference could introduce some heterogeneity and should be considered as a potential limitation. Nonetheless, given the biological consistency between the two indicators, it is unlikely that these differences in methodology could substantially change the overall findings. Lastly, it should be acknowledged that the recruitment periods for UBCS (2000–2001) and SPATZ (2012–2013) differed substantially. Although the pooled models included cohort adjustment to account for baseline differences, potential unmeasured temporal changes in lifestyle, diet or other factors may still have introduced residual heterogeneity and contribute to residual confounding.

## Conclusion

In conclusion, the study examined the association between child FUT2 secretor status and health outcomes in early life, focusing on mismatched secretor status in mother-infant pairs. Initial findings suggest potential links with BMI change in the first 4 months of life, but these associations were not statistically significant following adjustment for confounding factors. The study also highlights the potential mechanistic role of HMO exposure in discordant mother-infant pairs, where mismatched secretor status may influence microbial colonisation, nutrient utilisation, and immune development, with implication for early growth and infection susceptibility. Despite its limitations such as the predominance of secretor phenotypes in the study and small sample sizes of non-secretors, our approach provides valuable insights that may not be captured in studies focusing solely on typical secretor-to-secretor or non-secretor-to-non-secretor matches.

## Supplementary Information

Below is the link to the electronic supplementary material.

Supplementary Material 1

## Data Availability

Due to participant consent and data protection, we may not be able to share the raw data. However, the authors are open to sharing aggregate data, which have been included as Supplementary Materials.
