# Supplementary material for: Influence of infant fucosyltransferase polymorphisms on the association of maternal secretor status with child outcomes: the Ulm birth cohort studies
Source: Sci Rep. 2025 Nov 18;15:40339. doi: 10.1038/s41598-025-28217-w (PMC12627658; doi:10.1038/s41598-025-28217-w)
Supplement: Supplementary file 1 — Supplementary Material 1 [file 41598_2025_28217_MOESM1_ESM.docx]

**Influence of infant fucosyltransferase polymorphisms on the association of maternal secretor status with child outcomes: the Ulm Birth Cohort Studies**

Linda P. Siziba^1*^, Marko Mank^2^, Bernd Stahl^2,3^, Hermann Brenner^4^, John Gonsalves^2^, Bernadet Blijenberg^2^, Katrin Horn^5^, Markus Scholz^5^, Parastoo Kheiroddin^6^, Michael Kabesch^6^, Deborah Wernecke^7,8^, Dietrich Rothenbacher^7,8^ and Jon Genuneit^1^

^1^Pediatric Epidemiology, Department of Paediatrics, Medical Faculty, Leipzig University, 04103 Leipzig, Germany;

^2^Danone Research & Innovation, 3584 CT Utrecht, The Netherlands;

^3^Department of Chemical Biology & Drug Discovery, Utrecht Institute for Pharmaceutical Sciences, Utrecht University, 3584 Utrecht, The Netherlands

^4^Division of Clinical Epidemiology and Aging Research, German Cancer Research Centre (DKFZ), Im Neuenheimer Feld 581, 69120 Heidelberg, Germany

^5^Institute for Medical Informatics, Statistics and Epidemiology (IMISE), Medical Faculty, Leipzig University, Leipzig, Germany.

^6^Department of Pediatric Pneumology and Allergy, University Children's Hospital Regensburg (KUNO), of the University of Regensburg and the Order of St. John at the St. Hedwig Hospital, Regensburg, Germany.

^7^Institute of Epidemiology and Medical Biometry, Ulm University, 89075 Ulm, Germany

^8^German Center for Child and Adolescent Health (DZKJ), partner site Ulm, Ulm, Germany

*Correspondence: Linda.Siziba@medizin.uni-leipzig.de

**Supplementary Material**

**Table S1**: Demographic characteristics stratified by mother-child secretor status matches in the Ulm SPATZ Health Study (SPATZ)

|  |  | **Mother-Child secretor status match** | | | | | | | | | |  |
| --- | --- | --- | --- | --- | --- | --- | --- | --- | --- | --- | --- | --- |
|  | | secretor to secretor | | non-secretor to non-secretor | | secretor to non-secretor | | non-secretor to secretor | | non-secretor to mixed status* | |  |
|  |  | n | (% or mean) | n | (% or mean) | n | (% or mean) | n | (% or mean) | n | (% or mean) |  |
| Child sex | |  |  |  |  |  |  |  |  |  |  |  |
|  | Male | 196 | (54.6%) | 19 | (47.5%) | 26 | (50.0%) | 30 | (48.4%) | 16 | (61.5%) | 0.677 |
|  | Female | 163 | (45.4%) | 21 | (52.5%) | 26 | (50.0%) | 32 | (51.6%) | 10 | (38.5%) |  |
| Birth weight (g) | | 358 | 3380.0 | 40 | 3600.0 | 52 | 3182.5 | 62 | 3552.5 | 26 | 3530.0 | <0.001 |
| EBF | |  |  |  |  |  |  |  |  |  |  |  |
|  | Yes | 286 | (79.7%) | 32 | (80.0%) | 38 | (73.1%) | 46 | (74.2%) | 23 | (88.5%) | 0.492 |
|  | No | 73 | (20.3%) | 8 | (20.0%) | 14 | (26.9%) | 16 | (25.8%) | 3 | (11.5%) |  |
| AD up to 2 years | |  |  |  |  |  |  |  |  |  |  |  |
|  | Yes | 69 | (24.9%) | 8 | (25.8%) | 11 | (22.9%) | 12 | (23.5%) | 6 | (31.6%) | 0.961 |
|  | No | 208 | (75.1%) | 23 | (74.2%) | 37 | (77.1%) | 39 | (76.5%) | 13 | (68.4%) |  |
| OM up to 2 years | |  |  |  |  |  |  |  |  |  |  |  |
|  | Yes | 84 | (29.6%) | 12 | (36.4%) | 8 | (17.4%) | 12 | (22.6%) | 4 | (21.1%) | 0.270 |
|  | No | 200 | (70.4%) | 21 | (63.6%) | 38 | (82.6%) | 41 | (77.4%) | 15 | (78.9%) |  |
| LRTI up to 2 years | |  |  |  |  |  |  |  |  |  |  |  |
|  | Yes | 138 | (45.0%) | 17 | (51.5%) | 15 | (31.9%) | 29 | (53.7%) | 8 | (36.4%) | 0.190 |
|  | No | 169 | (55.0%) | 16 | (48.5%) | 32 | (68.1%) | 25 | (46.3%) | 14 | (63.6%) |  |
| URTI up to 2 years | |  |  |  |  |  |  |  |  |  |  |  |
|  | Yes | 242 | (79.3%) | 26 | (78.8%) | 39 | (83.0%) | 48 | (88.9%) | 17 | (81.0%) | 0.572 |
|  | No | 63 | (20.7%) | 7 | (21.2%) | 8 | (17.0%) | 6 | (11.1%) | 4 | (19.0%) |  |
| Maternal age (years) | | 359 | 32.6 (30.1; 35.9) | 40 | 33.7 (30.9; 36.2) | 52 | 34.3 (31.8; 36.4) | 61 | 33.8 (31.6; 36.3) | 26 | 31.4 (28.3; 35.9) | 0.052 |
| Parity (n births of foetus ≥ 24 weeks) | | |  |  |  |  |  |  |  |  |  |  |
|  | 0 births | 187 | (52.2%) | 20 | (50.0%) | 30 | (57.7%) | 28 | (45.2%) | 15 | (57.7%) | 0.691 |
|  | ≥ 1 birth | 171 | (47.8%) | 20 | (50.0%) | 22 | (42.3%) | 34 | (54.8%) | 11 | (42.3%) |  |
| Gestational age | |  |  |  |  |  |  |  |  |  |  |  |
|  | ≤ 36 | 20 | (5.6%) | 2 | (5.0%) | 6 | (11.5%) | 1 | (1.6%) | 1 | (3.8%) |  |
|  | Between 36 and 41 | 285 | (79.6%) | 34 | (85.0%) | 36 | (69.2%) | 51 | (82.3%) | 17 | (65.4%) |  |
|  | ≥ 41 | 53 | (14.8%) | 4 | (10.0%) | 10 | (19.2%) | 10 | (16.1%) | 8 | (30.8%) |  |
| Delivery mode | |  |  |  |  |  |  |  |  |  |  |  |
| Vaginal (spontaneous or assisted) | | 287 | (80.2%) | 30 | (75.0%) | 39 | (75.0%) | 50 | (80.6%) | 22 | (84.6%) | 0.783 |
| Caesarean (elective or emergency) | | 71 | (19.8%) | 10 | (25.0%) | 13 | (25.0%) | 12 | (19.4%) | 4 | (15.4%) |  |
| Maternal blood group | |  |  |  |  |  |  |  |  |  |  |  |
|  | A | 149 | (43.4%) | 19 | (48.7%) | 22 | (44.0%) | 21 | (33.9%) | 13 | (52.0%) |  |
|  | B | 45 | (13.1%) | 2 | (5.1%) | 9 | (18.0%) | 18 | (29.0%) | 0 | (0.0%) |  |
|  | O | 134 | (39.1%) | 15 | (38.5%) | 18 | (36.0%) | 21 | (33.9%) | 11 | (44.0%) |  |
|  | AB | 15 | (4.4%) | 3 | (7.7%) | 1 | (2.0%) | 2 | (3.2%) | 1 | (4.0%) |  |
| Rhesus Factor | |  |  |  |  |  |  |  |  |  |  |  |
|  | positive | 280 | (82.1%) | 35 | (89.7%) | 42 | (84.0%) | 50 | (80.6%) | 19 | (76.0%) | 0.655 |
|  | negative | 61 | (17.9%) | 4 | (10.3%) | 8 | (16.0%) | 12 | (19.4%) | 6 | (24.0%) |  |
| Maternal milk group | |  |  |  |  |  |  |  |  |  |  |  |
|  | I | 329 | (91.6%) | 0 | (0.0%) | 51 | (98.1%) | 0 | (0.0%) | 19 | (73.1%) |  |
|  | II | 0 | . | 37 | (92.5%) | 0 | (0.0%) | 58 | (93.5%) | 6 | (23.1%) |  |
|  | III | 30 | (8.4%) | 0 | . | 1 | (1.9%) | 0 | . | 1 | (3.8%) |  |
|  | IV | 0 | . | 3 | (7.5%) | 0 | (0.0%) | 4 | (6.5%) | 0 | . |  |

Sums (n) may not always add up to total because of missing values for certain items. Percentages exclude missing values. Maternal secretor status and milk groups were based on quantification and presence or absence of certain human milk oligosaccharides. Infant secretor status was based on the expression of three fucosyltransferase-2 (FUT2) single-nucleotide polymorphisms (SNPs), rs281379, rs516246 and rs602662. Secretors: homozygous wild type (SeSe) and/or heterozygous (Sese) genotypes for all three SNPs; non-secretors: homozygous mutant genotype (sese) for all three SNPs; Mixed status: homozygous wild type and/or heterozygous genotype in combination with a homozygous mutant genotype. EBF: exclusive breastfeeding; OM: otitis media; LRTI: lower respiratory tract infections; URTI: upper respiratory tract infections.

**Tables S2**: Demographic characteristics stratified by mother-child secretor status matches in the Ulm Birth Cohort Study (UBCS)

|  | | **Mother-Child secretor status match** | | | | | | | | | | |  | | |  |
| --- | --- | --- | --- | --- | --- | --- | --- | --- | --- | --- | --- | --- | --- | --- | --- | --- |
|  | | **secretor to  secretor** | | **non-secretor to non-secretor** | | **secretor to non-secretor** | | **non-secretor to secretor** | | **non-secretor to mixed status*** | |  | | |  |  |
|  |  | **n** | **(%)** | **n** | **(%)** | **n** | **(%)** | **n** | **(%)** | **n** | **(%)** | | | **p** | | |
| Child sex |  |  |  |  |  |  |  |  |  |  |  | | |  | | |
|  | Male | 137 | (46.8%) | 22 | (62.9%) | 17 | (43.6%) | 56 | (54.9%) | 11 | (57.9%) | | | 0.228 | | |
|  | Female | 156 | (53.2%) | 13 | (37.1%) | 22 | (56.4%) | 46 | (45.1%) | 8 | (42.1%) | | |  |  |  |
| Birth weight (g) | | 293 | 3375.0 | 35 | 3390.0 | 39 | 3330.0 | 102 | 3460.0 | 19 | 3490.0 | | | 0.523 | | |
| EBF at 6 weeks | |  |  |  |  |  |  |  |  |  |  | | |  | | |
|  | Yes | 286 | (97.6%) | 34 | (97.1%) | 38 | (97.4%) | 101 | (99.0%) | 19 | (100.0%) | | | 0.758 | | |
|  | No | 7 | (2.4%) | 1 | (2.9%) | 1 | (2.6%) | 1 | (1.0%) | 0 |  | | |  |  |  |
| AD diagnosis up to 2 years | |  |  |  |  |  |  |  |  |  |  | | |  | | |
|  | Yes | 70 | (35.4%) | 7 | (38.9%) | 10 | (37.0%) | 20 | (29.0%) | 3 | (25.0%) | | | 0.807 | | |
|  | No | 128 | (64.6%) | 11 | (61.1%) | 17 | (63.0%) | 49 | (71.0%) | 9 | (75.0%) | | |  |  |  |
| OM diagnosis up to 2 years | |  |  |  |  |  |  |  |  |  |  | | |  | | |
|  | Yes | 104 | (48.1%) | 10 | (43.5%) | 16 | (50.0%) | 38 | (49.4%) | 9 | (56.3%) | | | 0.954 | | |
|  | No | 112 | (51.9%) | 13 | (56.5%) | 16 | (50.0%) | 39 | (50.6%) | 7 | (43.8%) | | |  |  |  |
| LRTI diagnosis up to 2 years | |  |  |  |  |  |  |  |  |  |  | | |  | | |
|  | Yes | 196 | (78.4%) | 17 | (68.0%) | 28 | (80.0%) | 65 | (76.5%) | 13 | (76.5%) | | | 0.789 | | |
|  | No | 54 | (21.6%) | 8 | (32.0%) | 7 | (20.0%) | 20 | (23.5%) | 4 | (23.5%) | | |  |  |  |
| URTI diagnosis up to 2 years | |  |  |  |  |  |  |  |  |  |  | | |  | | |
|  | Yes | 98 | (38.1%) | 11 | (39.3%) | 15 | (40.5%) | 37 | (41.1%) | 7 | (41.2%) | | | 0.989 | | |
|  | No | 159 | (61.9%) | 17 | (60.7%) | 22 | (59.5%) | 53 | (58.9%) | 10 | (58.8%) | | |  |  |  |
| Maternal age (years) | | 293 | 32 | 35 | 31.9 | 39 | 31.3 | 101 | 33.9 | 19 | 32 | | | 0.058 | | |
| Parity (n births of foetus ≥ 24 weeks) | | |  |  |  |  |  |  |  |  |  | | |  | | |
|  | 0 births | 138 | (47.1%) | 17 | (48.6%) | 25 | (64.1%) | 45 | (44.1%) | 10 | (52.6%) | | | 0.295 | | |
|  | ≥ 1 birth | 155 | (52.9%) | 18 | (51.4%) | 14 | (35.9%) | 57 | (55.9%) | 9 | (47.4%) | | |  |  |  |
| Gestational age | |  |  |  |  |  |  |  |  |  |  | | |  | | |
|  | ≤ 36 | 8 | (2.7%) | 1 | (2.9%) | 2 | (5.1%) | 6 | (5.9%) | 1 | (5.6%) | | |  | | |
|  | Between 36 and 41 | 225 | (77.1%) | 26 | (76.5%) | 30 | (76.9%) | 71 | (69.6%) | 15 | (83.3%) | | |  | | |
|  | ≥ 41 | 59 | (20.2%) | 7 | (20.6%) | 7 | (17.9%) | 25 | (24.5%) | 2 | (11.1%) | | |  | | |
| Delivery mode | |  |  |  |  |  |  |  |  |  |  | | |  | | |
| Vaginal (spontaneous or assisted) | | 253 | (86.3%) | 24 | (68.6%) | 35 | (89.7%) | 85 | (83.3%) | 17 | (89.5%) | | | 0.094 | | |
| Caesarean (elective or emergency) | | 40 | (13.7%) | 11 | (31.4%) | 4 | (10.3%) | 17 | (16.7%) | 2 | (10.5%) | | |  |  |  |
| Maternal blood group | |  |  |  |  |  |  |  |  |  |  | | |  | | |
|  | A | 118 | (40.7%) | 14 | (40.0%) | 15 | (38.5%) | 42 | (41.2%) | 9 | (47.4%) | | |  | | |
|  | B | 32 | (11.0%) | 6 | (17.1%) | 4 | (10.3%) | 16 | (15.7%) | 0 | (0.0%) | | |  | | |
|  | AB | 8 | (2.8%) | 0 | (0.0%) | 0 | (0.0%) | 7 | (6.9%) | 3 | (15.8%) | | |  | | |
|  | O | 132 | (45.5%) | 15 | (42.9%) | 20 | (51.3%) | 37 | (36.3%) | 7 | (36.8%) | | |  | | |
| Rhesus Factor | |  |  |  |  |  |  |  |  |  |  | | |  | | |
|  | positive | 251 | (86.6%) | 28 | (80.0%) | 31 | (79.5%) | 84 | (82.4%) | 17 | (89.5%) | | | 0.527 | | |
|  | negative | 39 | (13.4%) | 7 | (20.0%) | 8 | (20.5%) | 18 | (17.6%) | 2 | (10.5%) | | |  |  |  |
| Lewis AB status | |  |  |  |  |  |  |  |  |  |  | | |  | | |
|  | A-B+ | 290 | (99.0%) | 0 | (0.0%) | 39 | (100.0%) | 0 | (0.0%) | 12 | (63.2%) | | |  | | |
|  | A+B- | 0 |  | 27 | (77.1%) | 0 |  | 45 | (44.1%) | 5 | (26.3%) | | |  | | |
|  | A-B- | 0 |  | 8 | (22.9%) | 0 |  | 57 | (55.9%) | 2 | (10.5%) | | |  | | |
|  | A+B+ | 3 |  | 0 |  | 0 |  | 0 |  | 0 |  | | |  | | |

Sums (n) may not always add up to total because of missing values for certain items. Percentages exclude missing values. Maternal secretor status based on Lewis blood group antigens (i.e. secretors: A-B+ or A+B+, non-secretors: A+B- or A-B-). Infant secretor status was based on the expression of three fucosyltransferase-2 (FUT2) single-nucleotide polymorphisms (SNPs), rs281379, rs516246 and rs602662. Secretors: homozygous wild type (SeSe) and/or heterozygous (Sese) genotypes for all three SNPs; non-secretors: homozygous mutant genotype (sese) for all three SNPs; Mixed status: homozygous wild type and/or heterozygous genotype in combination with a homozygous mutant genotype. EBF: exclusive breastfeeding; OM: otitis media; LRTI: lower respiratory tract infections; URTI: upper respiratory tract infections.

**Table S3**: Sample sizes for each subgroup included in the stratified analyses in the Ulm SPATZ Health Study

|  | | **AD (n)** | | **OM (n)** | | **LRTI (n)** | | **URTI (n)** | | **BMI measurements (n)** | | | |
| --- | --- | --- | --- | --- | --- | --- | --- | --- | --- | --- | --- | --- | --- |
| **Maternal status** | **Child status** | Yes | No | Yes | No | Yes | No | Yes | No | 4–5 weeks | 3-4 months | 6-7 months | 10-12 months |
| Secretor | Non-secretor | 11 | 37 | 8 | 38 | 15 | 32 | 39 | 8 | 52 | 51 | 50 | 49 |
|  | Secretor | 69 | 208 | 84 | 200 | 138 | 169 | 242 | 63 | 348 | 350 | 336 | 321 |
|  | Both | 5 | 10 | 4 | 11 | 7 | 11 | 14 | 3 | 19 | 19 | 15 | 15 |
| Non-secretor | Non-secretor | 8 | 23 | 12 | 21 | 17 | 16 | 26 | 7 | 39 | 40 | 39 | 38 |
|  | Secretor | 12 | 39 | 12 | 41 | 29 | 25 | 48 | 6 | 61 | 61 | 58 | 56 |
|  | Both | 1 | 3 | . | 4 | 1 | 3 | 3 | 1 | 6 | 6 | 5 | 5 |
| Secretor | Homozygous wildtype | 21 | 87 | 32 | 81 | 55 | 67 | 94 | 27 | 140 | 139 | 129 | 125 |
|  | Heterozygous | 40 | 105 | 47 | 99 | 74 | 86 | 132 | 27 | 180 | 184 | 177 | 168 |
|  | Homozygous mutant | 11 | 37 | 8 | 38 | 15 | 32 | 39 | 8 | 52 | 51 | 50 | 49 |
|  | Mixed | 13 | 26 | 9 | 31 | 16 | 27 | 30 | 12 | 47 | 46 | 45 | 43 |
| Non-secretor | Heterozygous | 12 | 38 | 12 | 40 | 28 | 25 | 47 | 6 | 59 | 59 | 56 | 55 |
|  | Homozygous mutant | 8 | 23 | 12 | 21 | 17 | 16 | 26 | 7 | 39 | 40 | 39 | 38 |
|  | Mixed | 1 | 4 | . | 5 | 2 | 3 | 4 | 1 | 8 | 8 | 7 | 6 |

Maternal secretor status and milk groups were based on quantification and presence or absence of certain human milk oligosaccharides. Infant secretor status was based on the expression of three fucosyltransferase-2 (FUT2) single-nucleotide polymorphisms (SNPs), rs281379, rs516246 and rs602662. Secretors: homozygous wild type (SeSe) and/or heterozygous (Sese) genotypes for all three SNPs; non-secretors: homozygous mutant genotype (sese) for all three SNPs; Mixed status: homozygous wild type and/or heterozygous genotype in combination with a homozygous mutant genotype. EBF: exclusive breastfeeding; AD: Atopic dermatitis; OM: otitis media; LRTI: lower respiratory tract infections; URTI: upper respiratory tract infections.

**Table S4**: Sample sizes for each subgroup included in the stratified analyses in the Ulm Birth Cohort Study

|  | | **AD (n)** | | **OM (n)** | | **LRTI (n)** | | **URTI (n)** | | **BMI measurements (n)** | | | |
| --- | --- | --- | --- | --- | --- | --- | --- | --- | --- | --- | --- | --- | --- |
| Maternal status | Child status | Yes | No | Yes | No | Yes | No | Yes | No | 4–5 weeks | 3-4 months | 6-7 months | 10-12 months |
| Secretor | Non-secretor | 10 | 17 | 16 | 16 | 28 | 7 | 15 | 22 | 38 | 39 | 39 | 38 |
|  | Secretor | 70 | 128 | 104 | 112 | 196 | 54 | 98 | 159 | 266 | 275 | 280 | 259 |
|  | Both | 3 | 5 | 6 | 4 | 8 | 3 | 5 | 6 | 12 | 12 | 12 | 11 |
| Non-secretor | Non-secretor | 7 | 11 | 10 | 13 | 17 | 8 | 11 | 17 | 32 | 33 | 32 | 31 |
|  | Secretor | 20 | 49 | 38 | 39 | 65 | 20 | 37 | 53 | 94 | 93 | 94 | 93 |
|  | Both | . | 4 | 3 | 3 | 5 | 1 | 2 | 4 | 7 | 7 | 7 | 6 |
| Secretor | Homozygous wildtype | 31 | 57 | 41 | 47 | 81 | 27 | 44 | 68 | 118 | 121 | 123 | 111 |
|  | Heterozygous | 33 | 61 | 54 | 56 | 102 | 22 | 45 | 81 | 129 | 134 | 137 | 129 |
|  | Homozygous mutant | 10 | 17 | 16 | 16 | 28 | 7 | 15 | 22 | 38 | 39 | 39 | 38 |
|  | Mixed | 9 | 15 | 15 | 13 | 21 | 8 | 14 | 16 | 31 | 32 | 32 | 30 |
| Non-secretor | Homozygous wildtype | 3 | 10 | 6 | 11 | 9 | 6 | 6 | 11 | 21 | 20 | 20 | 20 |
|  | Heterozygous | 16 | 36 | 27 | 26 | 50 | 14 | 27 | 40 | 66 | 66 | 67 | 66 |
|  | Homozygous mutant | 7 | 11 | 10 | 13 | 17 | 8 | 11 | 17 | 32 | 33 | 32 | 31 |
|  | Mixed | 1 | 7 | 8 | 5 | 11 | 1 | 6 | 6 | 14 | 14 | 14 | 13 |

Maternal secretor status based on Lewis blood group antigens (i.e. secretors: A-B+ or A+B+, non-secretors: A+B- or A-B-). Infant secretor status was based on the expression of three fucosyltransferase-2 (FUT2) single-nucleotide polymorphisms (SNPs), rs281379, rs516246 and rs602662. Secretors: homozygous wild type (SeSe) and/or heterozygous (Sese) genotypes for all three SNPs; non-secretors: homozygous mutant genotype (sese) for all three SNPs; Mixed status: homozygous wild type and/or heterozygous genotype in combination with a homozygous mutant genotype. AD: Atopic dermatitis; OM: otitis media; LRTI: lower respiratory tract infections; URTI: upper respiratory tract infections.

**
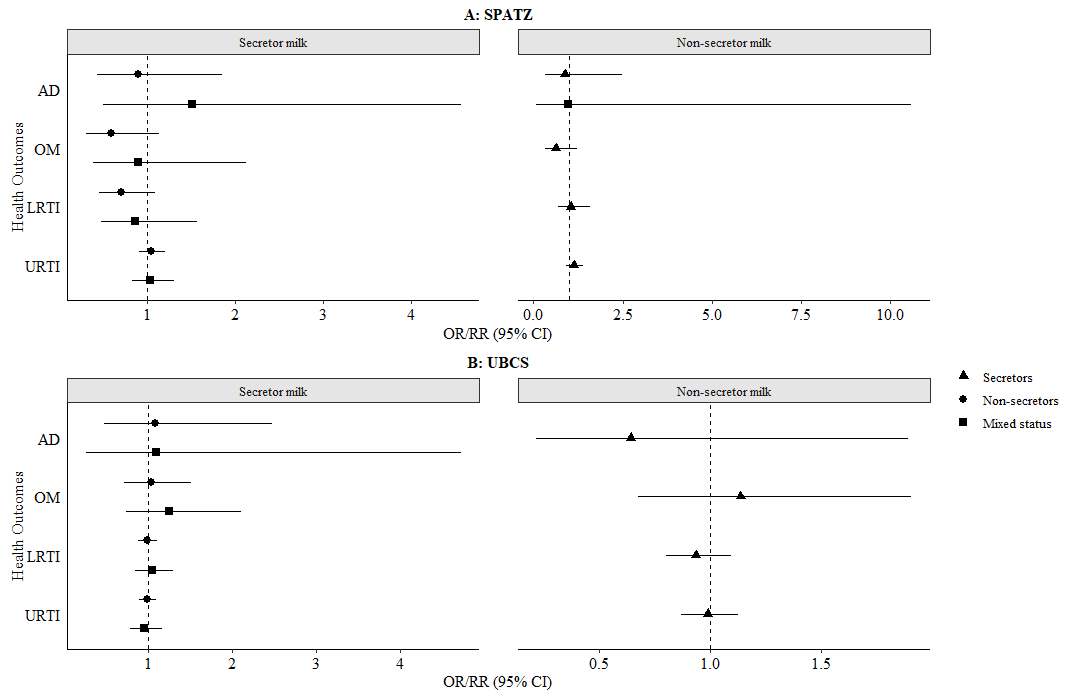
**

**Figure S1.** Crude associations of infant secretor status with health outcomes up to 2 years stratified by secretor and non-secretor milk in the Ulm Birth Cohorts. Secretor status was determined by three fucosyltransferase-2 (FUT2) SNPs (rs281379, rs516246, rs602662). Maternal secretor status (secretor or non-secretor milk) was assessed by human milk oligosaccharides in the SPATZ study and Lewis blood group antigens in the UBCS study. Infant genotypes were classified as: secretor (homozygous wild type, SeSe, or heterozygous, Sese, for all three SNPs), non-secretor (homozygous mutant, sese, for all three SNPs), or mixed (any combination of homozygous wild type, heterozygous, or homozygous mutant for any SNP). Associations with atopic dermatitis (AD) were analysed by logistic regression (odds ratios, OR), and infections (otitis media, OM; lower respiratory tract infections, LRTI; upper respiratory tract infections, URTI) by modified Poisson regression (risk ratios, RR). CI: Confidence Intervals; SPATZ: Ulm SPATZ Health Study; UBCS: Ulm Birth Cohort Study. Bonferroni-adjusted significance level: α = 0.05/14 = 0.0035


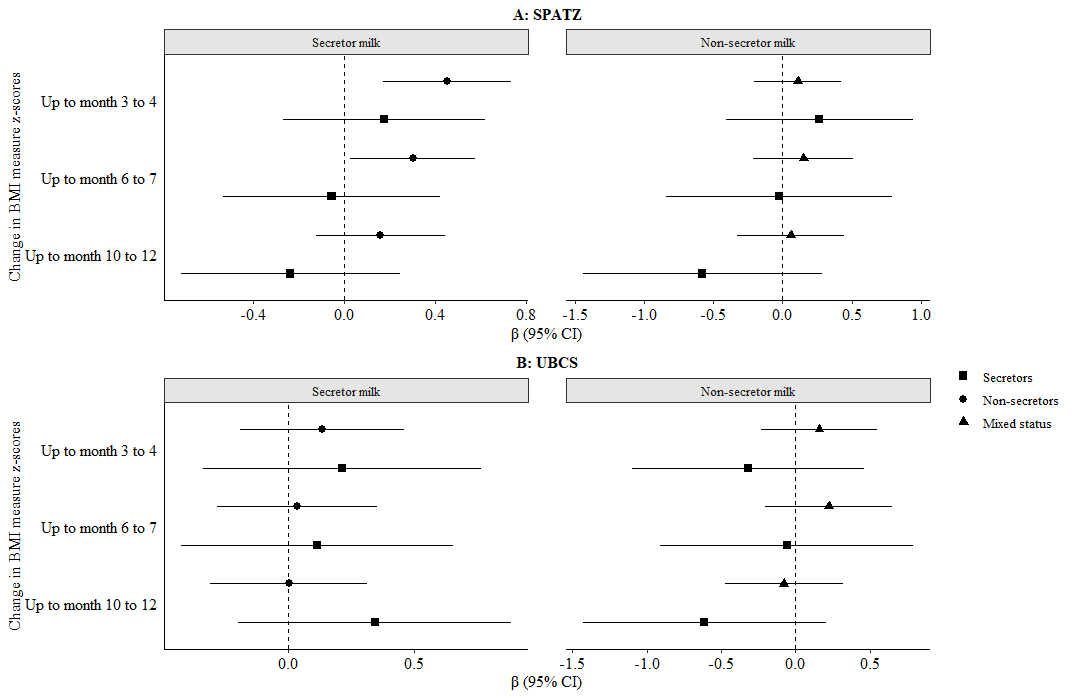


**Figure S2**. Crude associations between infant secretor status and body mass index (BMI) changes in the first year of life, stratified by maternal secretor and non-secretor milk in the Ulm Birth Cohorts. Infant secretor status was determined by three fucosyltransferase-2 (FUT2) SNPs (rs281379, rs516246, rs602662). Maternal secretor status was assessed by human milk oligosaccharides in SPATZ and Lewis blood group antigens in UBCS. Infants were classified as: secretor (homozygous wild type, SeSe, or heterozygous, Sese, for all three SNPs), non-secretor (homozygous mutant, sese, for all three SNPs), or mixed (any combination of homozygous wild type, heterozygous, or homozygous mutant for any SNP). Associations with infant BMI were analysed using general linear models, with BMI z-scores adjusted for sex and age between sampling periods. SPATZ: Ulm SPATZ Health Study; UBCS: Ulm Birth Cohort Study. Bonferroni-adjusted significance level: α = 0.05/14 = 0.0035.


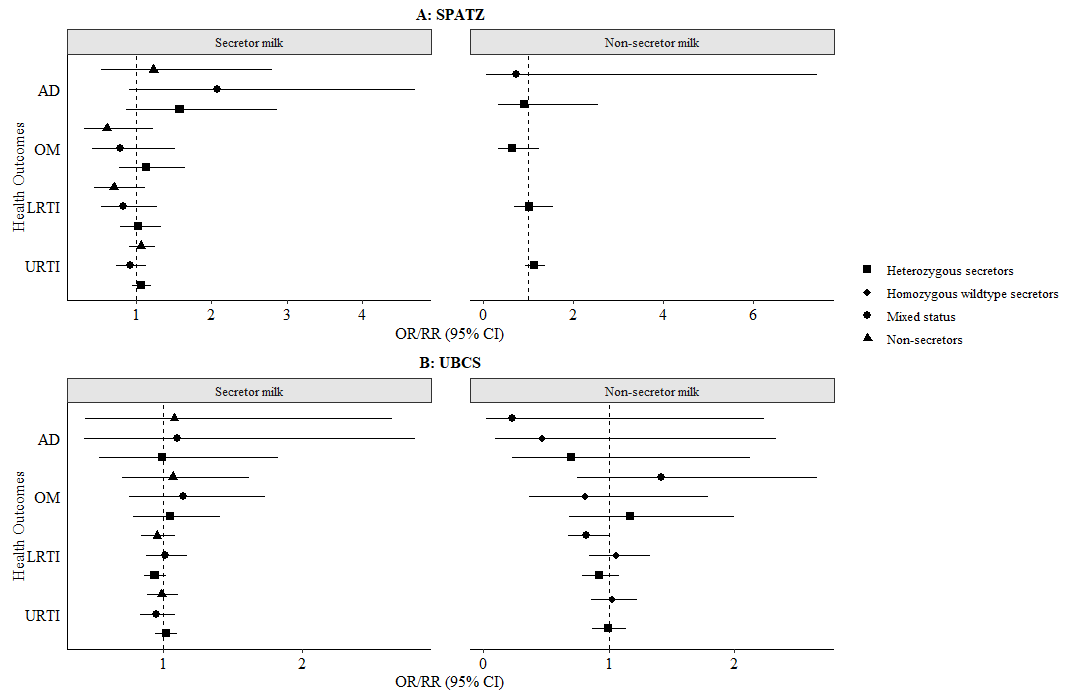


**Figure S3.** Crude associations between infant fucosyltransferase-2 (FUT2) heterozygosity and health outcomes in the first 2 years of life, stratified by maternal secretor and non-secretor milk in the Ulm Birth Cohorts. Maternal secretor status was determined by human milk oligosaccharides in SPATZ and Lewis blood group antigens in UBCS. Infant secretor status was based on three FUT2 SNPs (rs281379, rs516246, rs602662): homozygous wild type secretors (SeSe for all SNPs), heterozygous secretors (Sese for all SNPs), non-secretors (sese for all SNPs), or mixed (any combination of homozygous wild type, heterozygous, or homozygous mutant for any SNP). Associations with atopic dermatitis (AD) were analysed by logistic regression (odds ratios, OR), and infections (otitis media, OM; lower respiratory tract infections, LRTI; upper respiratory tract infections, URTI) by modified Poisson regression (risk ratios, RR). CI: Confidence Intervals; SPATZ: Ulm SPATZ Health Study; UBCS: Ulm Birth Cohort Study. Bonferroni-adjusted significance level: α = 0.05/14 = 0.0035.


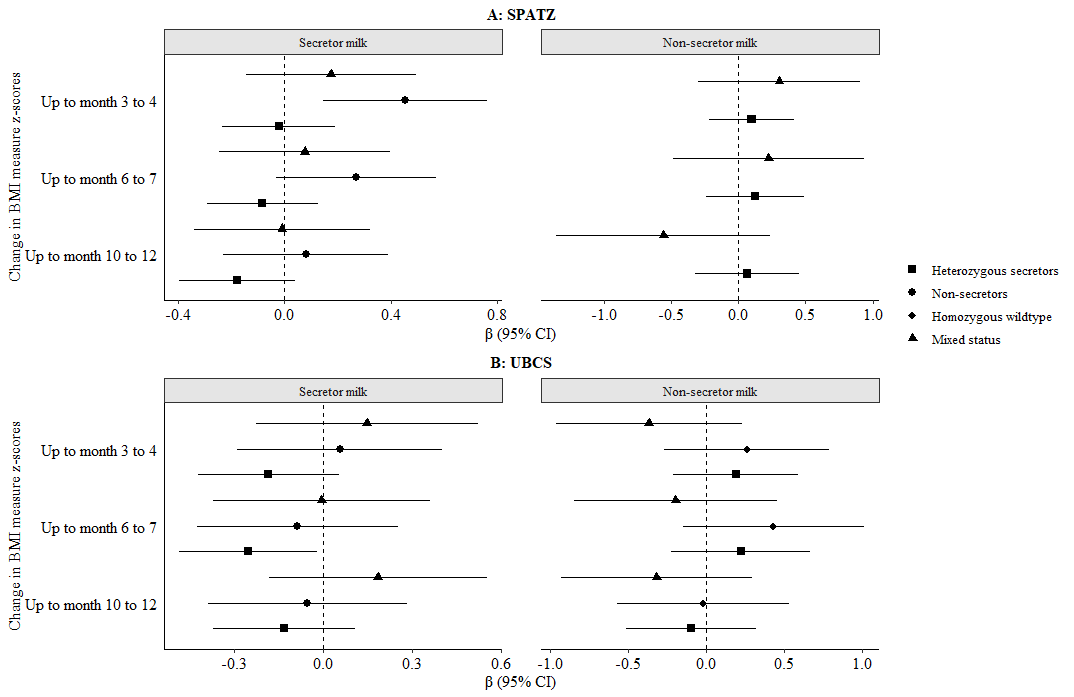


**Figure S4.** Crude associations between infant fucosyltransferase-2 (FUT2) heterozygosity and body mass index (BMI) changes in the first year of life, stratified by maternal secretor and non-secretor milk in the Ulm Birth Cohorts. Maternal secretor status was determined by human milk oligosaccharides in SPATZ and Lewis blood group antigens in UBCS. Infant secretor status was based on three FUT2 SNPs (rs281379, rs516246, rs602662): homozygous wild type secretors (SeSe for all SNPs), heterozygous secretors (Sese for all SNPs), non-secretors (sese for all SNPs), or mixed (any combination of homozygous wild type, heterozygous, or homozygous mutant for any SNP). Associations with BMI change, calculated as the difference between BMI at 4–5 weeks and BMI z-scores adjusted for sex and age, were analysed using general linear models. SPATZ: Ulm SPATZ Health Study; UBCS: Ulm Birth Cohort Study. Bonferroni-adjusted significance level: α = 0.05/14 = 0.0035.


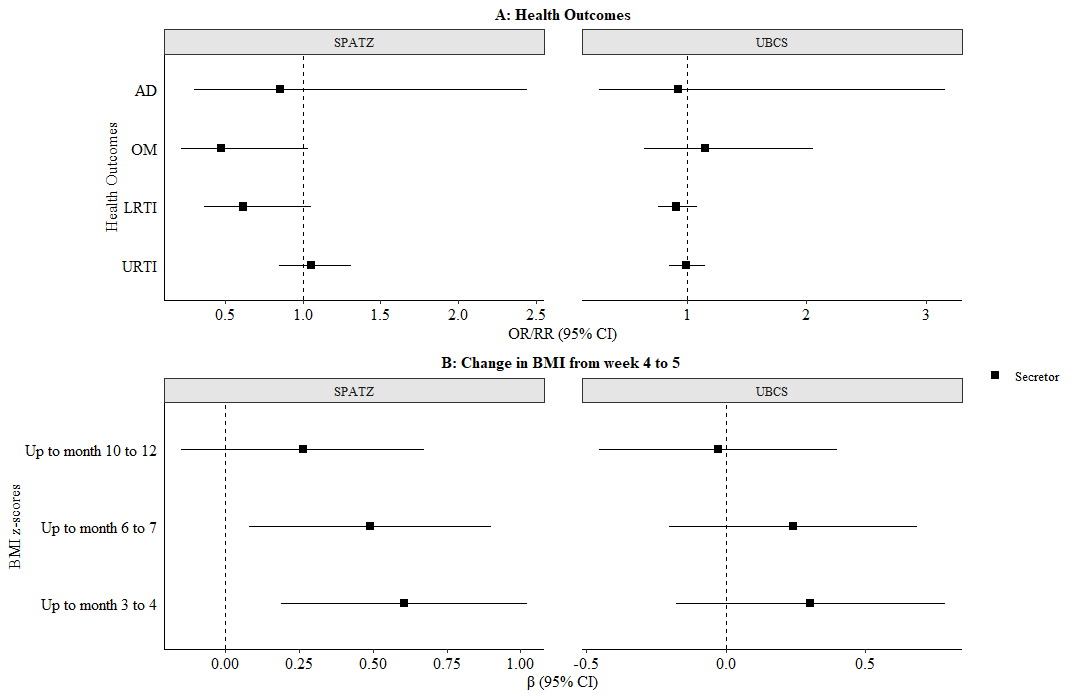


**Figure S5.** Crude associations of maternal secretor status with (A) health outcomes in the first year of life and (B) body mass index (BMI) changes in non-secretor infants, using non-secretor milk as the reference. Maternal secretor status was determined by human milk oligosaccharides in SPATZ and Lewis blood group antigens in UBCS. Infant non-secretor status was defined by homozygous mutant genotype (sese) for three FUT2 SNPs (rs281379, rs516246, rs602662). In (A), associations with atopic dermatitis (AD) were analysed by logistic regression (odds ratios, OR), and infections (otitis media, OM; lower respiratory tract infections, LRTI; upper respiratory tract infections, URTI) by modified Poisson regression (risk ratios, RR). In (B), associations with BMI change, calculated as the difference between BMI at 4–5 weeks and later time points, were analysed using general linear models, with BMI z-scores adjusted for sex and age. CI: Confidence Intervals; SPATZ: Ulm SPATZ Health Study; UBCS: Ulm Birth Cohort Study. Bonferroni-adjusted significance level: α = 0.05/14 = 0.0035.

**
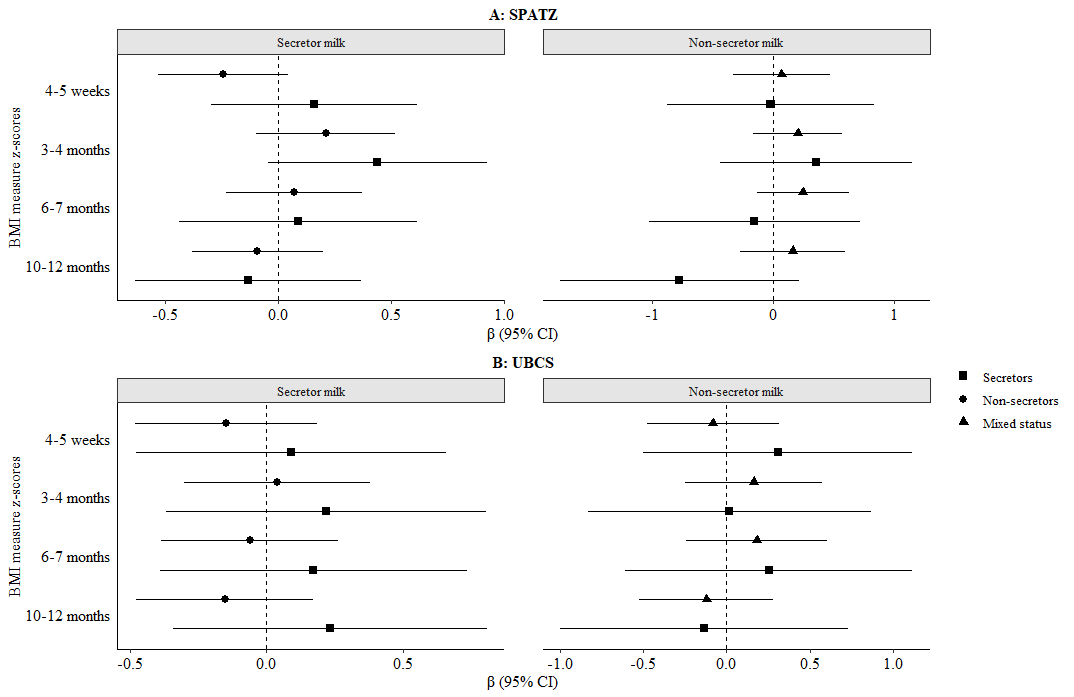
**

**Figure S6**. Crude associations between infant secretor status and body mass index (BMI) in the first year of life, stratified by maternal secretor and non-secretor milk in the Ulm Birth Cohorts. Infant secretor status was determined by three fucosyltransferase-2 (FUT2) SNPs (rs281379, rs516246, rs602662): secretors (homozygous wild type, SeSe, or heterozygous, Sese, for all SNPs), non-secretors (homozygous mutant, sese, for all SNPs), or mixed (any combination of homozygous wild type, heterozygous, or homozygous mutant for any SNP). Maternal secretor status was assessed by human milk oligosaccharides in SPATZ and Lewis blood group antigens in UBCS. Associations with infant BMI were analysed using general linear models, with BMI z-scores adjusted for sex and age. SPATZ: Ulm SPATZ Health Study; UBCS: Ulm Birth Cohort Study. Bonferroni-adjusted significance level: α = 0.05/16 = 0.0031.


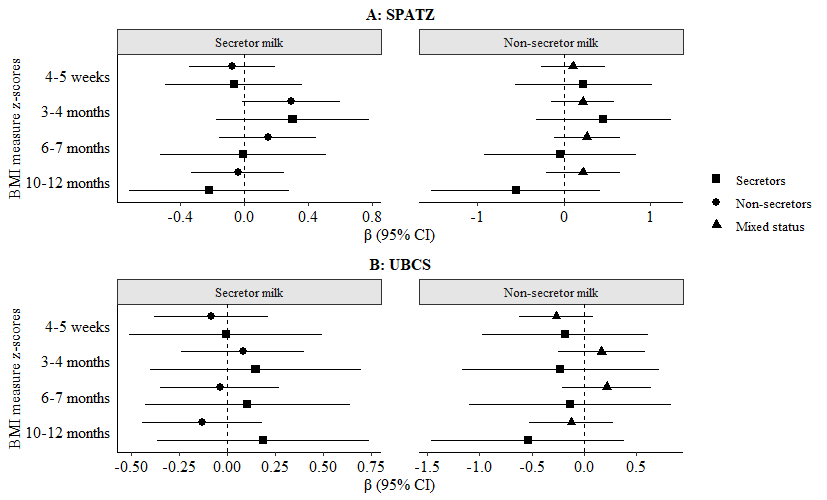


**Figure S7**. Adjusted associations between infant secretor status and body mass index (BMI) in the first year of life, stratified by maternal secretor and non-secretor milk in the Ulm Birth Cohorts. Infant secretor status was determined by three fucosyltransferase-2 (FUT2) SNPs (rs281379, rs516246, rs602662): secretors (homozygous wild type, SeSe, or heterozygous, Sese, for all SNPs), non-secretors (homozygous mutant, sese, for all SNPs), or mixed (any combination of homozygous wild type, heterozygous, or homozygous mutant for any SNP). Maternal secretor status was assessed by human milk oligosaccharides in SPATZ and Lewis blood group antigens in UBCS. Associations with infant BMI were analysed using general linear models, with BMI z-scores adjusted for sex, age, maternal Lewis and ABO blood groups, gestational age (weeks), and birthweight. SPATZ: Ulm SPATZ Health Study; UBCS: Ulm Birth Cohort Study. Bonferroni-adjusted significance level: α = 0.05/16 = 0.0031.


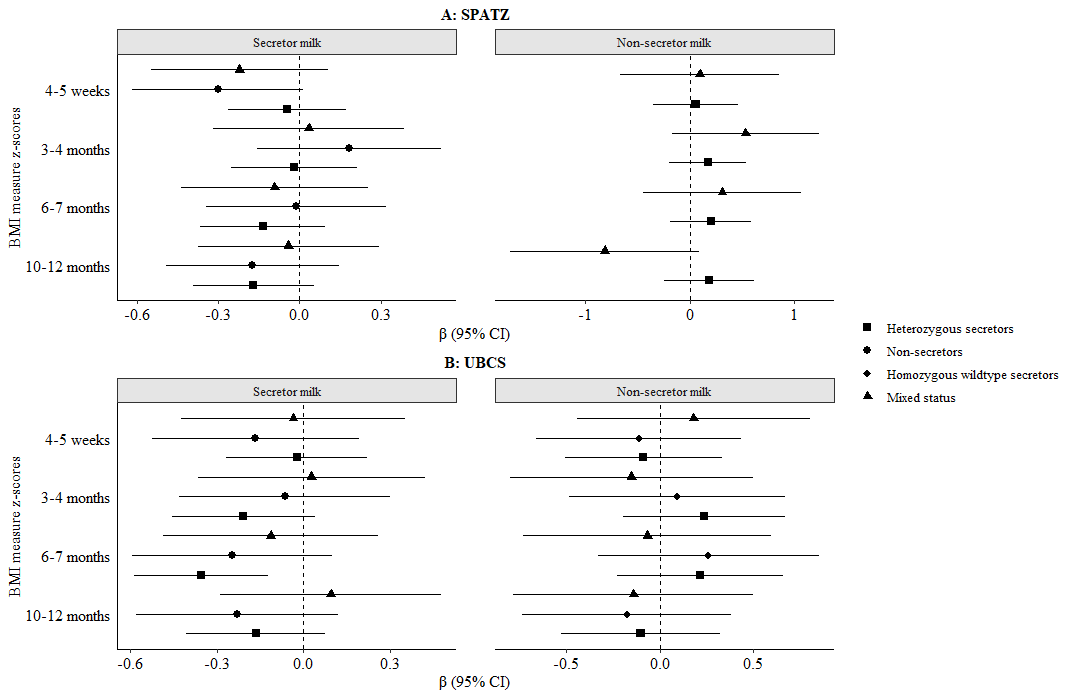


**Figure S8**. Crude associations between infant fucosyltransferase-2 (FUT2) heterozygosity and body mass index (BMI) changes in the first year of life, stratified by maternal secretor and non-secretor milk in the Ulm Birth Cohorts. Maternal secretor status was determined by human milk oligosaccharides in SPATZ and Lewis blood group antigens in UBCS. Infant secretor status was based on three FUT2 SNPs (rs281379, rs516246, rs602662): homozygous wild type secretors (SeSe for all SNPs), heterozygous secretors (Sese for all SNPs), non-secretors (sese for all SNPs), or mixed (any combination of homozygous wild type, heterozygous, or homozygous mutant for any SNP). Associations with BMI change, calculated as the difference between BMI at 4–5 weeks and BMI z-scores adjusted for sex and age, were analysed using general linear models. SPATZ: Ulm SPATZ Health Study; UBCS: Ulm Birth Cohort Study. Bonferroni-adjusted significance level: α = 0.05/16 = 0.0031.


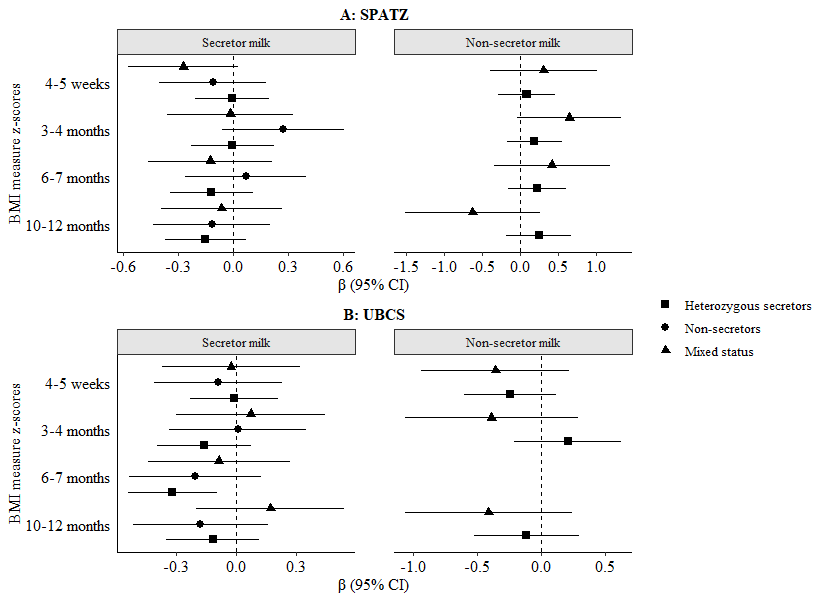


**Figure S9**. Adjusted associations between infant fucosyltransferase-2 (FUT2) heterozygosity and body mass index (BMI) changes in the first year of life, stratified by maternal secretor and non-secretor milk in the Ulm Birth Cohorts. Maternal secretor status was determined by human milk oligosaccharides in SPATZ and Lewis blood group antigens in UBCS. Infant secretor status was based on three FUT2 SNPs (rs281379, rs516246, rs602662): homozygous wild type secretors (SeSe for all SNPs), heterozygous secretors (Sese for all SNPs), non-secretors (sese for all SNPs), or mixed (any combination of homozygous wild type, heterozygous, or homozygous mutant for any SNP). Associations with BMI change were analysed using general linear models, with BMI z-scores adjusted for sex, age, maternal Lewis and ABO blood groups, gestational age (weeks), and birthweight. SPATZ: Ulm SPATZ Health Study; UBCS: Ulm Birth Cohort Study. Bonferroni-adjusted significance level: α = 0.05/16 = 0.0031.


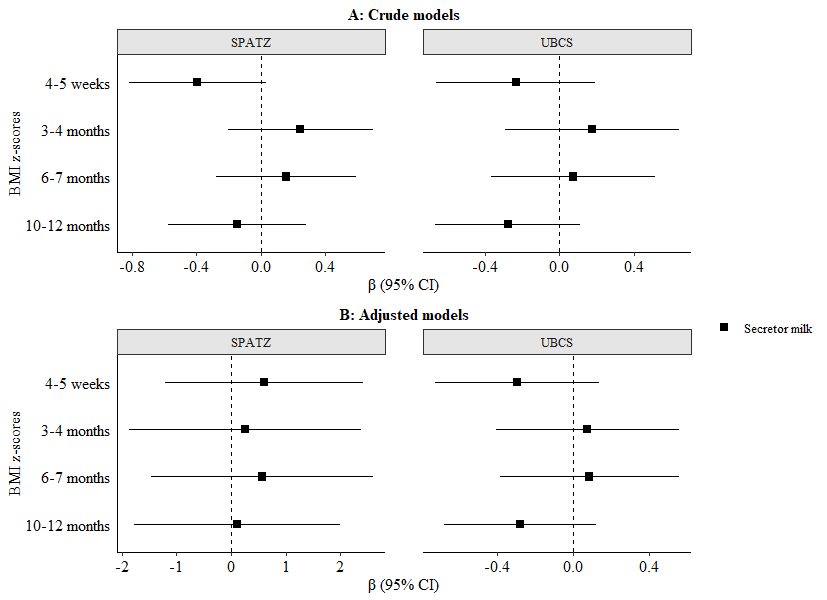


**Figure S10**. Crude (A) and adjusted (B) associations between maternal secretor status and infant body mass index (BMI) in the first year of life among non-secretor infants, with non-secretor milk as the reference. Maternal secretor status was determined by human milk oligosaccharides in SPATZ and Lewis blood group antigens in UBCS. Infant non-secretor status was defined by homozygous mutant genotype (sese) for three FUT2 SNPs (rs281379, rs516246, rs602662). Associations with BMI change were analysed using general linear models, with BMI z-scores adjusted for sex and age. Adjusted models (B) included maternal Lewis and ABO blood groups, gestational age (weeks), and birthweight. SPATZ: Ulm SPATZ Health Study; UBCS: Ulm Birth Cohort Study. Bonferroni-adjusted significance level: α = 0.05/16 = 0.0031.

**Table S5**: Pooled adjusted associations of infant secretor status with health outcomes up to 2 years stratified by secretor and non-secretor milk in the Ulm Birth Cohorts.

|  | | | **Secretor milk** | | **Non-secretor milk** | |
| --- | --- | --- | --- | --- | --- | --- |
| **Model** | **Outcome** | **Child FUT2 status** | **OR/RR (95% CI)*** | **p value** | **OR/RR (95% CI) *** | **p value** |
| 1 | AD | Mixed status | 1.34 (0.55, 3.26) | 0,52 | 0.31 (0.03, 2.76) | 0,29 |
|  |  | Non-secretors | 0.97 (0.56, 1.67) | 0,91 | Ref | |
|  |  | Secretors | Ref | | 0.77 (0.37, 1.63) | 0,50 |
|  | OM | Mixed status | 1.10 (0.69, 1.75) | 0,69 | 0.68 (0.26, 1.75) | 0,42 |
|  |  | Non-secretors | 0.82 (0.58, 1.15) | 0,24 | Ref | |
|  |  | Secretors | Ref | | 0.89 (0.61, 1.32) | 0,58 |
|  | LRTI | Mixed status | 0.97 (0.76, 1.23) | 0,79 | 0.81 (0.56, 1.16) | 0,24 |
|  |  | Non-secretors | 0.91 (0.78, 1.05) | 0,18 | Ref | |
|  |  | Secretors | Ref | | 0.97 (0.82, 1.15) | 0,71 |
|  | URTI | Mixed status | 0.98 (0.85, 1.14) | 0,83 | 1.02 (0.81, 1.29) | 0,87 |
|  |  | Non-secretors | 1.01 (0.93, 1.10) | 0,76 | Ref | |
|  |  | Secretors | Ref | | 1.04 (0.93, 1.16) | 0,50 |
| 2 | AD | Heterozygous | 1.26 (0.82, 1.92) | 0,29 | 0.81 (0.38, 1.73) | 0,59 |
|  |  | Homozygous wild type | Ref | | 0.54 (0.12, 2.38) | 0,41 |
|  |  | Mixed status | 1.55 (0.84, 2.86) | 0,16 | 0.37 (0.07, 1.92) | 0,24 |
|  |  | Non-secretors | 1.13 (0.62, 2.06) | 0,69 | Ref | |
|  | OM | Heterozygous | 1.10 (0.87, 1.39) | 0,44 | 0.91 (0.60, 1.36) | 0,63 |
|  |  | Homozygous wild type | Ref | | 0.65 (0.31, 1.35) | 0,25 |
|  |  | Mixed status | 0.99 (0.69, 1.42) | 0,96 | 0.96 (0.54, 1.70) | 0,88 |
|  |  | Non-secretors | 0.85 (0.59, 1.23) | 0,39 | Ref | |
|  | LRTI | Heterozygous | 0.97 (0.87, 1.07) | 0,50 | 0.96 (0.80, 1.14) | 0,62 |
|  |  | Homozygous wild type | Ref | | 1.09 (0.87, 1.37) | 0,47 |
|  |  | Mixed status | 0.94 (0.80, 1.11) | 0,50 | 0.83 (0.65, 1.06) | 0,13 |
|  |  | Non-secretors | 0.89 (0.76, 1.04) | 0,13 | Ref | |
|  | URTI | Heterozygous | 1.04 (0.97, 1.11) | 0,26 | 1.04 (0.93, 1.17) | 0,48 |
|  |  | Homozygous wild type | Ref | | 1.07 (0.90, 1.27) | 0,45 |
|  |  | Mixed status | 0.94 (0.83, 1.05) | 0,25 | 0.97 (0.80, 1.18) | 0,77 |
|  |  | Non-secretors | 1.03 (0.93, 1.12) | 0,60 | Ref | |

Infant secretor status was determined by three fucosyltransferase-2 (FUT2) SNPs (rs281379, rs516246, rs602662). Model 1: Secretors (homozygous wild type, SeSe, or heterozygous, Sese, for all SNPs); non-secretors (homozygous mutant, sese, for all SNPs). Model 2: Homozygous wild type secretors (SeSe for all SNPs), heterozygous secretors (Sese for all SNPs), non-secretors (sese for all SNPs), or mixed (any combination of homozygous wild type, heterozygous, or homozygous mutant for any SNP). Maternal secretor status was assessed by human milk oligosaccharides in SPATZ and Lewis blood group antigens in UBCS. Associations with atopic dermatitis (AD) were analysed by logistic regression (odds ratios, OR), and infections (otitis media, OM; lower respiratory tract infections, LRTI; upper respiratory tract infections, URTI) by modified Poisson regression (risk ratios, RR). Models were adjusted for study and birthweight (infections only). CI: Confidence Intervals; SPATZ: Ulm SPATZ Health Study; UBCS: Ulm Birth Cohort Study. Bonferroni-adjusted significance level: α = 0.05/14 = 0.0035.

**Table S6:** Pooled adjusted associations of infant secretor status with infant body mass index (BMI) in the first year of life stratified by secretor and non-secretor milk in the Ulm Birth Cohorts.

|  | | | **Secretor milk** | | **Non-secretor milk** | |
| --- | --- | --- | --- | --- | --- | --- |
| **Model** | **BMI measurement** | **Child FUT2 status** | **ß (95% CI)** | **p value** | **ß (95% CI)** | **p value** |
| 1 | 4-5 weeks | Mixed status | -0.03 (-0.36, 0.30) | 0,8487 | 0.13 (-0.41, 0.67) | 0,64 |
|  |  | Non-secretor | -0.08 (-0.28, 0.12) | 0,4199 | Ref | |
|  |  | Secretor | Ref | | -0.10 (-0.36, 0.16) | 0,46 |
|  | 3-4 months | Mixed status | 0.27 (-0.10, 0.64) | 0,1460 | 0.16 (-0.42, 0.74) | 0,58 |
|  |  | Non-secretor | 0.20 (-0.03, 0.42) | 0,0866 | Ref | |
|  |  | Secretor | Ref | | 0.15 (-0.13, 0.43) | 0,29 |
|  | 6-7 months | Mixed status | 0.07 (-0.31, 0.46) | 0,7075 | 0.09 (-0.52, 0.71) | 0,77 |
|  |  | Non-secretor | 0.06 (-0.16, 0.28) | 0,6002 | Ref | |
|  |  | Secretor | Ref | | 0.20 (-0.09, 0.48) | 0,18 |
|  | 10-12 months | Mixed status | -0.03 (-0.41, 0.34) | 0,8679 | -0.42 (-1.07, 0.22) | 0,20 |
|  |  | Non-secretor | -0.08 (-0.30, 0.13) | 0,4472 | Ref | |
|  |  | Secretor | Ref | | -0.02 (-0.31, 0.28) | 0,91 |
| 2 | 4-5 weeks | Heterozygous | -0.02 (-0.17, 0.13) | 0,7588 | -0.08 (-0.35, 0.19) | 0,54 |
|  |  | Homozygous mutant | -0.12 (-0.33, 0.10) | 0,2947 | Ref | |
|  |  | Homozygous wild type | Ref | | -0.14 (-0.61, 0.33) | 0,56 |
|  |  | Mixed status | -0.20 (-0.43, 0.03) | 0,0820 | 0.07 (-0.37, 0.52) | 0,74 |
|  | 3-4 months | Heterozygous | -0.10 (-0.27, 0.07) | 0,2484 | 0.17 (-0.11, 0.46) | 0,23 |
|  |  | Homozygous mutant | 0.14 (-0.11, 0.38) | 0,2710 | Ref | |
|  |  | Homozygous wild type | Ref | | 0.08 (-0.43, 0.58) | 0,77 |
|  |  | Mixed status | -0.00 (-0.26, 0.25) | 0,9786 | 0.06 (-0.40, 0.53) | 0,79 |
|  | 6-7 months | Heterozygous | -0.23 (-0.39, -0.07) | 0,0056 | 0.20 (-0.10, 0.50) | 0,18 |
|  |  | Homozygous mutant | -0.07 (-0.31, 0.17) | 0,5527 | Ref | |
|  |  | Homozygous wild type | Ref | | 0.26 (-0.26, 0.78) | 0,33 |
|  |  | Mixed status | -0.13 (-0.38, 0.12) | 0,3219 | 0.05 (-0.44, 0.55) | 0,83 |
|  | 10-12 months | Heterozygous | -0.16 (-0.32, 0.00) | 0,0510 | 0.01 (-0.29, 0.32) | 0,93 |
|  |  | Homozygous mutant | -0.16 (-0.40, 0.07) | 0,1726 | Ref | |
|  |  | Homozygous wild type | Ref | | -0.14 (-0.67, 0.39) | 0,61 |
|  |  | Mixed status | -0.01 (-0.26, 0.24) | 0,9542 | -0.37 (-0.89, 0.15) | 0,16 |

Infant secretor status was determined by three FUT2 SNPs (rs281379, rs516246, rs602662). Model 1: Secretors (homozygous wild type, SeSe, or heterozygous, Sese, for all SNPs); non-secretors (homozygous mutant, sese, for all SNPs); mixed (any combination of homozygous wild type, heterozygous, or homozygous mutant for any SNP). Model 2: Homozygous wild type secretors (SeSe for all SNPs), heterozygous secretors (Sese for all SNPs), non-secretors (sese for all SNPs), or mixed (as in Model 1). Maternal secretor status was assessed by human milk oligosaccharides in SPATZ and Lewis blood group antigens in UBCS. Associations with infant BMI were analysed using general linear models, with BMI z-scores adjusted for sex, age, maternal Lewis and ABO blood groups, gestational age (weeks), and birthweight. SPATZ: Ulm SPATZ Health Study; UBCS: Ulm Birth Cohort Study. Bonferroni-adjusted significance level: α = 0.05/16 = 0.0031.

**Table S7**: Pooled adjusted associations of infant fucosyltransferase-2 (FUT2) secretor status and heterozygosity with change in infant body mass index (BMI) in the first year of life stratified by secretor and non-secretor milk in the Ulm Birth Cohorts

|  | | | **Secretor milk** | | **Non-secretor milk** | |
| --- | --- | --- | --- | --- | --- | --- |
| **Model** | **BMI change from 4 to 5 week measure** | **Child FUT2 status** | **ß (95% CI)** | **p value** | **ß (95% CI)** | **p value** |
| 1 | Up to 3-4 months | Mixed status | 0.27 (-0.07, 0.61) | 0,11 | -0.02 (-0.53, 0.49) | 0,93 |
|  |  | Non-secretor | 0.25 (0.04, 0.46) | 0,02 | Ref | |
|  |  | Secretor | Ref | | 0.19 (-0.06,0.43) | 0,14 |
|  | Up to 6-7 months | Mixed status | 0.12 (-0.23, 0.47) | 0,49 | -0.07 (-0.63, 0.50) | 0,82 |
|  |  | Non-secretor | 0.10 (-0.10, 0.31) | 0,31 | Ref | |
|  |  | Secretor | Ref | | 0.24 (-0.02, 0.51) | 0,07 |
|  | Up to 10-12 months | Mixed status | 0.10 (-0.25, 0.45) | 0,58 | -0.56 (-1.14, 0.02) | 0,06 |
|  |  | Non-secretor | 0.03 (-0.17, 0.24) | 0,75 | Ref | |
|  |  | Secretor | Ref | | 0.04 (-0.23, 0.31) | 0,76 |
| 2 | Up to 3-4 months | Heterozygous | -0.10 (-0.26, 0.06) | 0,21 | 0.19 (-0.06, 0.44) | 0,14 |
|  |  | Homozygous mutant | 0.21 (-0.01, 0.44) | 0,07 | Ref | |
|  |  | Homozygous wild type | Ref | | 0.31 (-0.13, 0.76) | 0,17 |
|  |  | Mixed status | 0.19 (-0.05, 0.43) | 0,12 | -0.07 (-0.48, 0.35) | 0,75 |
|  | Up to 6-7 months | Heterozygous | -0.18 (-0.33, -0.03) | 0,02 | 0.22 (-0.05, 0.50) | 0,11 |
|  |  | Homozygous mutant | 0.02 (-0.20, 0.24) | 0,86 | Ref | |
|  |  | Homozygous wild type | Ref | | 0.48 (-0.00, 0.96) | 0,05 |
|  |  | Mixed status | 0.07 (-0.16, 0.30) | 0,56 | -0.02 (-0.47, 0.44) | 0,94 |
|  | Up to 10-12 months | Heterozygous | -0.17 (-0.32, -0.01) | 0,03 | 0.03 (-0.24,0.31) | 0,82 |
|  |  | Homozygous mutant | -0.04 (-0.26, 0.18) | 0,72 | Ref | |
|  |  | Homozygous wild type | Ref | | 0.08 (-0.40, 0.55) | 0,75 |
|  |  | Mixed status | 0.11 (-0.13, 0.35) | 0,36 | -0.30 (-0.77, 0.16) | 0,20 |

Infant secretor status was determined by three fucosyltransferase-2 (FUT2) SNPs (rs281379, rs516246, rs602662). Model 1: Secretors (homozygous wild type, SeSe, or heterozygous, Sese, for all SNPs); non-secretors (homozygous mutant, sese, for all SNPs); mixed (any combination of homozygous wild type, heterozygous, or homozygous mutant for any SNP). Model 2: Homozygous wild type secretors (SeSe for all SNPs), heterozygous secretors (Sese for all SNPs), non-secretors (sese for all SNPs), or mixed (as in Model 1). Maternal secretor status was assessed by human milk oligosaccharides in SPATZ and Lewis blood group antigens in UBCS. Associations with BMI change, calculated as the difference from BMI at 4–5 weeks, were analysed using general linear models, with BMI z-scores adjusted for sex, age, study, and birthweight. β: Beta estimate; CI: Confidence Intervals; SPATZ: Ulm SPATZ Health Study; UBCS: Ulm Birth Cohort Study. Bonferroni-adjusted significance level: α = 0.05/14 = 0.0035.

**Table S8**: Pooled adjusted associations of maternal secretor status with health outcomes in the first two years of life, infant body mass index (BMI) and change in BMI amongst non-secretor infants.

|  | **Outcome** | **OR/RR (95% CI)*** | **p value** |
| --- | --- | --- | --- |
| **Health outcomes** | AD | 0.88 (0.40, 1.96) | 0, 76 |
|  | OM | 0.78 (0.49, 1.24) | 0, 29 |
|  | LRTI | 0.82 (0.67, 1.01) | 0, 06 |
|  | URTI | 1.04 (0.92, 1.18) | 0, 54 |
|  |  | **ß (95% CI)** | **p value** |
| **BMI measure at** | 4-5 weeks | -0.20 (-0.50, 0.09) | 0, 17 |
|  | 3-4 months | 0.23 (-0.10, 0.56) | 0, 17 |
|  | 6-7 months | 0.13 (-0.19, 0.45) | 0, 42 |
|  | 10-12 months | -0.21 (-0.50, 0.09) | 0, 17 |
| **Change in BMI from 4 to 5 weeks** | Up to 3-4 months | 0.38 (0.07, 0.69) | 0, 02 |
|  | Up to 6-7 months | 0.28 (-0.02, 0.58) | 0, 07 |
|  | Up to 10-12 months | 0.07 (-0.22, 0.36) | 0, 63 |

Infant secretor status was determined by three FUT2 SNPs (rs281379, rs516246, rs602662), with non-secretor infants having a homozygous mutant genotype (sese) for all SNPs. Maternal secretor status was assessed by human milk oligosaccharides in SPATZ and Lewis blood group antigens in UBCS, with non-secretor milk as the reference. Associations with atopic dermatitis (AD) were analysed by logistic regression (odds ratios, OR), infections (otitis media, OM; lower respiratory tract infections, LRTI; upper respiratory tract infections, URTI) by modified Poisson regression (risk ratios, RR), and BMI/change in BMI by general linear models. BMI change was calculated as the difference from BMI at 4–5 weeks, with z-scores adjusted for sex and age. Models were adjusted for study and birthweight (BMI models). CI: Confidence Intervals; SPATZ: Ulm SPATZ Health Study; UBCS: Ulm Birth Cohort Study. Bonferroni-adjusted significance level: α = 0.05/14 = 0.0035.
